# SIRT6-dependent cysteine monoubiquitination in the PRE-SET domain of Suv39h1 regulates the NF-κB pathway

**DOI:** 10.1038/s41467-017-02586-x

**Published:** 2018-01-09

**Authors:** Irene Santos-Barriopedro, Laia Bosch-Presegué, Anna Marazuela-Duque, Carolina de la Torre, Carlota Colomer, Berta N. Vazquez, Thomas Fuhrmann, Bárbara Martínez-Pastor, Wenfu Lu, Thomas Braun, Eva Bober, Thomas Jenuwein, Lourdes Serrano, Manel Esteller, Zhenbang Chen, Silvia Barceló-Batllori, Raúl Mostoslavsky, Lluis Espinosa, Alejandro Vaquero

**Affiliations:** 1 Chromatin Biology Laboratory, Cancer Epigenetics and Biology Program (PEBC), Bellvitge Biomedical Research Institute (IDIBELL), Av. Gran Via de l’Hospitalet 199-203, 08908 L’Hospitalet de Llobregat (Barcelona), Spain; 2 Tissue Repair and Regeneration Group, Department of Biosciences, Universitat de Vic, Universitat Central de Catalunya, Barcelona, 08500 Vic Spain; 3 Proteomics Unit, Bellvitge Biomedical Research Institute (IDIBELL), Av. Gran Via de l’Hospitalet 199-203, 08908 L’Hospitalet de Llobregat (Barcelona), Spain; 4 Program in Cancer Research, IMIM-Hospital del Mar, Barcelona Biomedical Research Park (PRBB), Barcelona, 08003 Spain; 5 Human Genetics Institute of New Jersey, Rutgers, The State University of New Jersey, 145 Bevier Road, Piscataway, NJ 08854 USA; 6 Department of Epigenetics, Max Planck Institute of Immunobiology and Epigenetics, Stübeweg 51, 79108 Freiburg, Germany; 7 Massachusetts General Hospital Cancer Center, Harvard Medical School, Boston, MA 02114 USA; 8 Department of Biochemistry and Cancer Biology, Meharry Medical College, Nashville, TN 37208 USA; 9 Department of Cardiac Development and Remodelling, Max-Planck-Institute for Heart and Lung Research, Bad Nauheim, D-61231 Germany; 10 Cancer Epigenetics Laboratory, Cancer Epigenetics and Biology Program (PEBC), Bellvitge Biomedical Research Institute (IDIBELL), Av. Gran Via de l’Hospitalet 199-203, 08908 L’Hospitalet de Llobregat (Barcelona), Spain; 11 Department of Physiological Sciences II, School of Medicine, University of Barcelona, Barcelona, 08907 Catalonia Spain; 12 Institució Catalana de Recerca i Estudis Avançats (ICREA), Barcelona, 08010 Catalonia Spain

## Abstract

Sirtuins are NAD^+^-dependent deacetylases that facilitate cellular stress response. They include SirT6, which protects genome stability and regulates metabolic homeostasis through gene silencing, and whose loss induces an accelerated aging phenotype directly linked to hyperactivation of the NF-κB pathway. Here we show that SirT6 binds to the H3K9me3-specific histone methyltransferase Suv39h1 and induces monoubiquitination of conserved cysteines in the PRE-SET domain of Suv39h1. Following activation of NF-κB signaling Suv39h1 is released from the IκBα locus, subsequently repressing the NF-κB pathway. We propose that SirT6 attenuates the NF-κB pathway through IκBα upregulation via cysteine monoubiquitination and chromatin eviction of Suv39h1. We suggest a mechanism based on SirT6-mediated enhancement of a negative feedback loop that restricts the NF-κB pathway.

## Abstract (web summary)

Sirtuins are involved in the regulation of responses to diverse types of cellular stress. Here the authors describe the SirT6-dependent cysteine monoubiquitination of the histone methyltransferase Suv39h1 as part of a regulatory circuit for the NF-κB pathway.

## Introduction

Adaptation to stress is a major survival challenge at the cellular and organism levels. The Sir2 proteins (or sirtuins) are major coordinators of cellular response to diverse types of stress, such as genotoxic, oxidative, and metabolic stress^1,2^. They perform their regulatory roles through NAD^+^-dependent deacetylation of histone and non-histone proteins^3–5^. A second enzymatic activity, mono[ADP-ribosyl]transferase (ADPRT) activity, has been also detected in some sirtuins^6–8^, although the functional implications of this dual activity are not well understood. There are seven mammalian sirtuins, named SirT1–7. Among the most important roles performed by sirtuins is to preserve genome stability, which they do at many levels, including regulation of chromatin structure, cell cycle control, gene expression, DNA replication, and DNA repair^9^. One of the best described examples is the functional relationship between SirT1 and Suv39h1, the principal mammalian H3K9 histone methyltransferase (HMT)^10^. Suv39h1 regulates the structure of both constitutive heterochromatin (CH) and facultative heterochromatin (FH) regions by depositing H3K9me3, and through interplay with heterochromatin-specific factors such as HP1 proteins^11,12^. Under stress conditions SirT1 promotes FH formation in specific genomic regions (e.g., rDNA regions) in a coordinated sequence of events, including recruitment of Suv39h1 and subsequent activation of it through deacetylation of K266 in its catalytic SET domain^13,14^. In contrast, under oxidative stress, SirT1 promotes protection of CH structures by stabilizing Suv39h1^15^.

SirT6 is, together with SirT1, one of the sirtuins most clearly involved in maintaining genome stability. SirT6 exhibits both enzymatic activities (deacetylase and ADPRT), although the former seems to be predominant^7,16,17^. SirT6 has been shown to deacetylate histone and non-histone proteins (e.g., CtIP)^18^. In the case of histones, SirT6 targets mainly two histone H3 marks, H3K9Ac and H3K56Ac^16,19,20^. H3K9Ac is involved in gene silencing and regulation of chromatin structure, whereas H3K56Ac participates in DNA damage signaling^21,22^. Loss of SirT6 in mice induces a phenotype that resembles accelerated aging: it includes high levels of genome instability and myriad metabolic defects, including low serum glucose and IGF-1, lymphopenia, loss of subcutaneous fat, and lordokyphosis^23^. SirT6 has been involved in three major functions, all of which it executes via chromatin regulation. First, SirT6 controls genome stability by regulating telomere structure and by participating in DNA repair^16,18,24–26^. Second, it also has an important role in glucose and lipid homeostasis^27–29^. Third, SirT6 apparently serves a protective role by inhibiting expression of a specific set of genes involved in critical pathways, such as the hypoxia factor HIF1α^30^, and the master regulator of stress response and inflammation nuclear factor-κB (NF-κB). The functional link between SirT6 and NF-κB seems to be very relevant in vivo, as suggested by the finding that the progeria phenotype of *sirt6*^−/−^ mice is at least partly caused by hyperactivation of the NF-κB pathway^31^.

NF-κB is a family of five proteins (RelA, RelB, c-Rel, p50, and p52) that form homodimeric or heterodimeric complexes that function as transcription factors. The family members regulate cellular response to many internal or external stimuli including apoptosis inhibition, cell cycle control and proliferation, cell adhesion, tissue remodeling, inflammatory response, immunological adaptation, and the circadian clock^32–34^. Based on the mechanism of activation, the NF-κB pathway can be divided into two forms: the canonical pathway and non-canonical pathway^35,36^. The general regulatory mechanism of the canonical pathway involves sequestering of the NF-κB factors in the cytoplasm by direct interaction with the general inhibitors of NF-κB (or *I*κ*Bs*), the best studied of which is IκBα. Upon activation of the pathway, the IKK kinase complex (comprising IKKα, β, and γ) phosphorylates IκBα, inducing its degradation and promoting the release and relocalization of NF-κB (RelA/p50) to the nucleus, where it exerts its role in gene expression^32^. Suv39h1 has been shown to localize to some NF-κB-regulated genes and repress their expression^37,38^. Upon tumor necrosis factor-α (TNFα) activation, IKKα localizes to the promoter and phosphorylates H3S10^39,40^, which in turn correlates with the loss of Suv39h1 in the same region^37^.

Interestingly, SirT1 and SirT2 have also been involved in NF-κB regulation. Both have been shown to deacetylate RelA in K310 inhibiting its transcriptional capacity^41,42^. As for SirT6, evidence suggests that its main role is to attenuate hyperactive NF-κB. Thus, upon activation of the pathway and arrival of NF-κB to the target genes, SirT6 localizes to the promoters of these genes, deacetylates H3K9Ac and consequently, promotes silencing of the target genes^31^. However, no additional information is currently available on the functions, mechanism or signaling partners of SirT6 in this repression.

Seeking to understand the role of SirT6 in epigenetic silencing, we have identified a link between SirT6 and Suv39h1, where SirT6 induces cysteine monoubiquitination (mUb) of Suv39h1 PRE-SET through the E3 ubiquitin ligase SKP2 in the context of NF-κB pathway activation. We report that loss of Suv39h1 in 293F cells or in wild-type (*wt*) mouse embryonic fibroblasts (MEFs) attenuates the general response to TNFα treatment at the gene-expression level, except for certain critical genes including the general repressor IκBα. Based on our results, we propose a novel mechanism whereby, in addition to attenuating NF-κB-mediated transcription activation gene-by-gene, SirT6 also promotes global inhibition of the pathway by reinforcing IκBα expression.

## Results

### SirT6 co-elutes with the H3K9-specific HMTs Suv39h1 and G9a

**Fig. 1:**
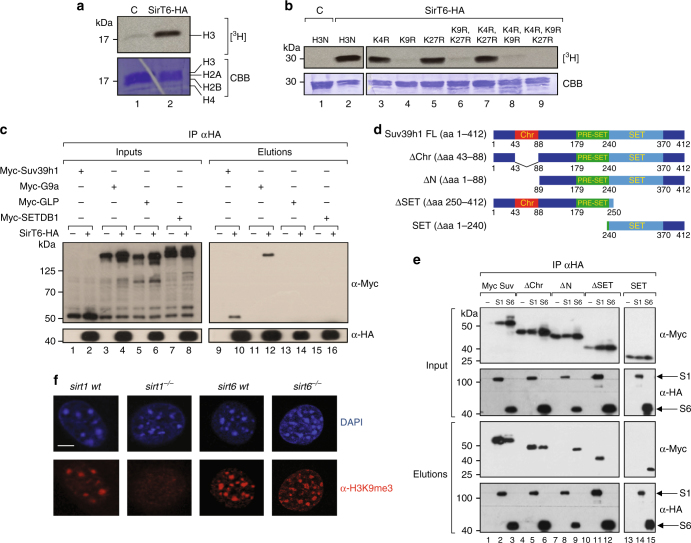
SirT6 co-elutes with the H3K9me-specific HMTs Suv39h1 and G9a. **a** In vitro HMT assay of SirT6-HA using [^3^H]-labeled SAM and core histones as substrates. HA-affinity purification of extracts from 293F cells transfected with either empty vector (C) or SirT6-HA. As a loading control, a Coomassie blue-staining of the PVDF membrane is shown (CBB). **b** HMT assay performed as in **a**, but using, as substrate, purified recombinant proteins formed by GST fused to the N-terminal H3 histone tail (with the indicated mutations). GST-fused N-terminal H3 histone tails proteins were stained with CBB. **c** HA immunoprecipitation of extracts from 293F cells transfected with SirT6-HA together with each of the nuclear H3K9 HMTs. **d** Schematics of the different Myc-tagged constructs of Suv39h1 used in **e**. **e** HA immunoprecipitation of extracts from 293F cells transfected with SirT6-HA and the indicated Myc-tagged mutants of Suv39h1. **f** Immunofluorescence analysis of H3K9me3 and DAPI in wild type (*wt*), *sirt1*^−/−^, and *sirt6*^−/−^ MEFs. A representative 5 μm scale bar is included in DAPI *sirt1* *wt* image

Aiming to understand the role of SirT6 in chromatin regulation and considering the close link between histone deacetylation and methylation, we first decided to determine whether SirT6 co-fractionates with an HMT activity that could provide clues on said function. Thus, we purified SirT6-HA from 293F cells and tested the elution in an in vitro HMT assay. We clearly observed that a histone H3-specific HMT activity was present in purified SirT6 fractions (Fig. 1a). To determine the specific residue(s) targeted by this HMT activity, we performed the in vitro HMT assay with purified GST (glutathione *S*-transferase)-fusion proteins containing the histone H3 N-terminal tail (aa1–28), either as WT, or as mutants in which we replaced one or more (in different combinations) of the main lysine residues methylated in histone H3 with arginine residues^43^. The results indicated that the HMT activity specifically targeted K9 in histone H3 (Fig. 1b).

We next aimed to identify the HMT related to SirT6. We employed immunoprecipitation experiments to determine whether SirT6 interacts with any of the principal H3K9-specific HMTs (Suv39h1, G9a, GLP, and SETDB1). Two of these enzymes, Suv39h1 and G9a, interacted with SirT6 (Fig. 1c). Although Suv39h1 is a known partner of SirT1^13^, several evidence strongly suggested a distinctive relationship between SirT6 and Suv39h1. First, SirT6-Suv39h1 interaction was not mediated by SirT1, as co-immunoprecipitation experiments showed no interaction between SirT1 and SirT6 (Supplementary Figure 1a). Second, using different deletion mutants of Suv39h1 (Fig. 1d, and Supplementary Figure 1b and 1c), we determined that in contrast to SirT1, which interacts with Suv39h1 through the N-terminal region (Fig. 1e, lane 8)^13^, SirT6 specifically binds to a the last 172 residues of Suv39h1 containing the C-terminal catalytic SET domain (aa 240–370) and POST-SET domain (aa 370-412) (Fig. 1e, lane 15, and Supplementary Figure 1b, c). Loss of the POST-SET domain (SETΔC) decreased significantly the capacity of the SET domain to bind to SirT6, suggesting that this region of the protein is also involved in the interaction (Supplementary Figure 1b, c). Third, H3K9me3 was completely lost in in pericentric heterochromatin of *sirt1*^−/−^ MEFs but was unaffected in *sirt6*^−/−^ MEFs (Fig. 1f).

### SirT6 induces a posttranslational modification in Suv39h1

**Fig. 2:**
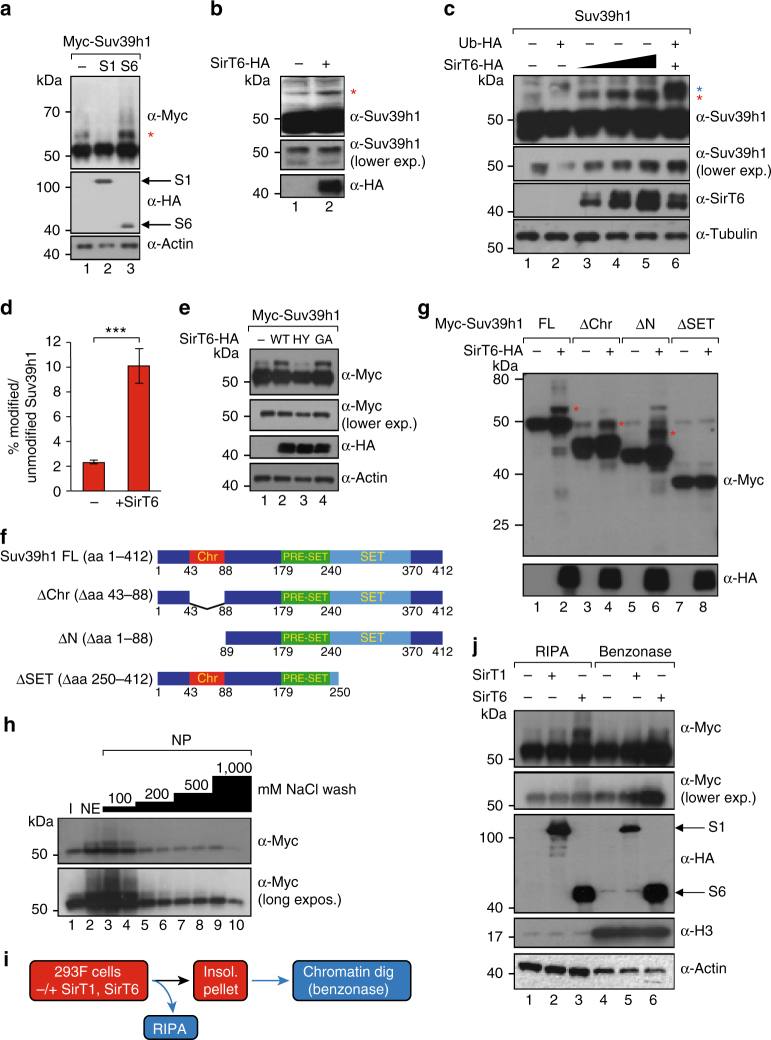
SirT6 induces a modification in Suv39h1. **a** Western blotting of extracts from 293F cells transfected with Myc-Suv39h1 in the presence or absence of HA-tagged SirT1 or SirT6 (lanes 2 and 3, respectively). A SirT6-induced modification in Suv39h1 is indicated (red asterisk). **b** Endogenous Suv39h1 is also modified upon SirT6 upregulation. Suv39h1 Western-blot of extracts from 293 cells overexpressed or not with SIRT6-HA. **c** Western blotting of extracts from 293F cells expressing non-tagged Suv39h1 in the presence or absence of either Ubiquitin-HA^15^ and/or SirT6-HA. The effect of a titration of SirT6-HA (1, 3 and 6 μg transfected) on Suv39h1 (2 μg transfected) was tested (lanes 3–5). A lower exposition of the Suv39h1 main band is also shown. Red and blue asterisks indicate endogenous or HA-tagged ubiquitination in Suv39h1, respectively. **d** Quantification of the levels of modified Myc-Suv39h1 in the absence or presence SirT6-HA expression. Relative levels (%) of Suv39h1 modification compared to unmodified Suv39h1 are shown. The results were obtained from *n* = 3 replicas of experiment shown in lanes 1–2 of Fig. 4c. **e** Analysis, as in **a**, of Myc-Suv39h1 cotransfected with different HA-tagged SirT6 mutants. WT: SirT6 wild type; HY: H133Y; GA: G60A. **f** Schematics of the different Myc-tagged constructs of Suv39h1 used in **g**. **g** Western blotting with the indicated different Myc-Suv39h1 constructs − / + SirT6-HA. **h** Fractionation of 293F cells co-transfected with Myc-Suv39h1 and SirT6-HA. Nuclear extracts (NE) and nuclear insoluble pellet (NP) were generated using the Dignam method. NP was step-washed with increasing concentrations of NaCl (from 100–1000 mM). **i** Schematic summary of the experiment shown in **j**. **j** Fractionation of 293 cells transfected with Myc-Suv39h1 and − / + SirT1 or SirT6. Fractionation with the RIPA method generated a soluble fraction (RIPA, lanes 1–3) and a NP, which was further digested with Benzonase (lanes 4–6)

In the previous experiments, we realized that overexpression of SirT6, but not of SirT1, induced an 8–10 kDa modification in both exogenous myc-tagged and endogenous Suv39h1 (Fig. 2a, b, red asterisk). Short hairpin RNA (shRNA)-driven downregulation of Suv39h1 confirmed that the observed band corresponded to endogenous modified Suv39h1 (Supplementary Figure 2a). The modification was not only SirT6-dependent (Fig. 2c)—but that it also required the enzymatic activity of SirT6, as the catalytically dead point-mutant SirT6 H133Y (HY) could not induce the modification (Fig. 2e, lane 3). A SirT6 mutant with inactive ADP-ribosylation activity G60A (GA) induced the modification just as WT SirT6 did, suggesting the deacetylation activity of SirT6 is involved (Fig. 2e, lane 4). We estimated that the population of modified Myc-Suv39h1 represents a 3% of unmodified Myc-Suv39h1 (Fig. 2d). SirT6 overexpression increased the levels of the modification to around 9% of unmodified Suv39h1. Endogenous modified Suv39h1 was significantly less abundant. Although we could not generate a reliable quantification, we roughly estimated that the endogenous modification was around 10 times less abundant than Myc-Suv39h1 modification. The ability of SirT6 to induce the modification seemed to depend on cell type as it was detected in 293F, H1299, HCT116, and NIH3T3 but not in HeLa or U2OS cells (Supplementay Figure 2b). We also confirmed that induction of the modification by SirT6 required direct binding to Suv39h1 as loss of the SET domain in Suv39h1 completely abrogated the ability of SirT6 to induce the band (Fig. 2f, g, red asterisks).

Finally, two experiments provided important evidence on the modification. First, the modification was present mainly in nuclear extract, and the fraction that remained bound to the chromatin pellet was washed away in low stringency conditions (Fig. 2h). Second, RIPA buffer extraction revealed that the SirT6-induced modified Suv39h1 was present almost exclusively in the soluble fraction, and that the digested chromatin insoluble pellet, did not contain a significant amount of it (Fig. 2i, j). Altogether, our findings strongly suggested that the modification does not affect Suv39h1 in the more compacted and insoluble chromatin regions, leading us to consider that it might actually be a mechanism for evicting Suv39h1 from chromatin.

### Suv39h1 PRE-SET domain is ubiquitinated in cysteines

**Fig. 3:**
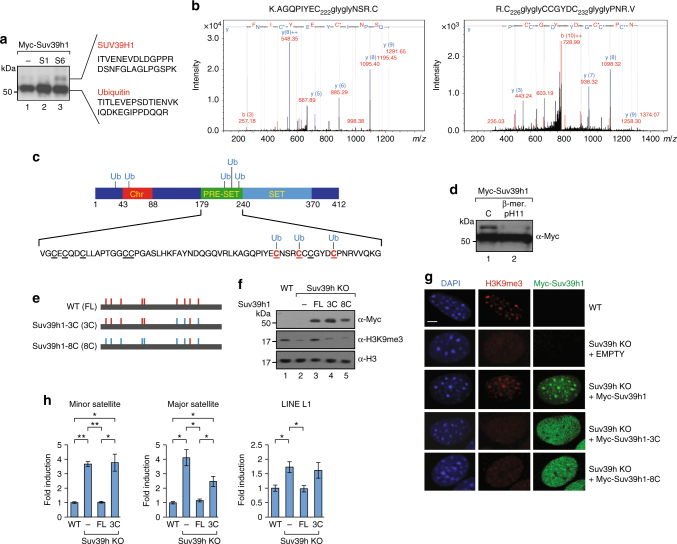
Monoubiquitination of cysteines in Suv39h1. **a** Suv39h1 from 293F cells transfected with SirT6-HA affinity-purified with myc resin and analyzed by mass spectrometry rendered peptides of Suv39h1 and ubiquitin. **b** MS/MS spectra corresponding to ubiquitinated peptides detected. Left panel: ubiquitinated peptide K.AGQPIYECNSR.C precursor *m*/*z* 676.3091 containing the Gly–Gly modification in cysteine 222. Right panel: ubiquitinated peptide R.CCCGYDCPNR.V precursor *m*/*z* 738,254 containing the Gly–Gly modification in cysteines 226 and 228. Corresponding MS/MS spectra of the non-ubiquitinated precursors and tables are shown in Figure 2. Additional data are shown in Supplementary Data Set 1. **c** Schematic representation of the monoubiquitination identified in Suv39h1. Primary sequence of the PRE-SET domain is indicated. The nine conserved cysteines that define the PRE-SET domain are shown underlined. **d** Western blotting of Myc-Suv39h1-containing extracts incubated in the presence or absence of β-mercaptoethanol at pH11 (see Online Methods). **e** Schematic representations of Suv39h1 mutants 3C and 8C in the PRE-SET domain used in **f**–**h**. The vertical bars correspond to the nine conserved cysteines (in red). The cysteines mutated to alanine are shown as blue bars. **f** Global H3K9me3 levels in Suv39h1/2 double KO MEFs (Suv39h KO) cells rescued with empty vector, or equivalent levels of Myc-Suv39h1 full-length (FL), 3C, or 8C (lanes 2–5, respectively). WT MEFs were included as positive control (lane 1). Total histone H3 is used as control. **g** Immunofluorescence analysis of DAPI, H3K9me3, and Myc in the same rescue experiment performed in **f**. A representative 5 μm scale bar is included in DAPI WT image. **h** Quantitative RT-PCR analysis (*n* = 5) of the mRNA levels of minor and major satellites and LINE L1 in the same cells used in (f) (*T*-test; SEM, *p < 0.05, **p < 0.01)

We sought to identify exactly how SirT6 modifies Suv39h1. As SirT6-dependent modification involves an 8–10 kDa increase in Suv39h1 molecular weight, we reasoned that it might involve ubiquitin or a related protein (e.g. SUMO or Nedd8). Supporting this hypothesis, hemagglutinin (HA)-tagged Ubiquitin was incorporated into Suv39h1 upon SirT6 overexpression (Fig. 2c, lane 6). To confirm that ubiquitination was involved, we immunopurified Myc-Suv39h1 in the presence of SirT6, excised the modified Suv39h1 band and analyzed it by mass spectrometry (MS). The analysis revealed peptides from Suv39h1 and ubiquitin, strongly supporting that SirT6 had induced mUb of Suv39h1 (Suv39h1mUb) (Fig. 3a, Supplementary Figure 3, and Supplementary Data Set 1).

Next, we performed further MS  analysis (see Supplemental Methods) to identify the modified residue(s) in Suv39h1. Specifically, we were searching for signs of Gly–Gly, the hallmark of ubiquitin after trypsin digestion^44^. Strikingly, we did not detect any Gly–Gly signature associated to a lysine residue, but did find four associated to cysteine residues (C49, C222, C226, and C232), plus one, to serine (S29) (Fig. 3b, c). We used another experiment to confirm that Suv39h1 mUb had not occurred at any lysine residues (amide bond): incubation of monoubiquitinated Suv39h1 under strong reducing conditions at pH11 led to cleavage of the ubiquitin (Fig. 3d)^45^. Interestingly, of the four cysteine residues that we identified, three (C222, C226, and C232) were located in the PRE-SET domain of Suv39h1; in fact, they are among the nine conserved residues that define the domain.

Armed with the aforementioned findings, we decided to focus on mUb of the PRE-SET domain. Although the role of this domain is not clear, it has been suggested that it might participate in binding of Suv39h1 to DNA^46^, which would make it, together with H3K9me3 and HP1^47,48^, a determinant in the ability of Suv39h1 to bind to chromatin. Altogether, our data suggest that the real target of the Suv39h1 mUb induced by SirT6 is not a single residue, but rather the entire PRE-SET domain.

### Loss of PRE-SET cysteine residues impairs Suv39h1 function

Considering the results shown in Fig. 2h–j, we reasoned that mUb of conserved cysteine residues in Suv39h1 promoted by SirT6 deactivates the PRE-SET domain specifically. This in turn could interfere with the ability of Suv39h1 to bind to chromatin and consequently, would trigger eviction of the deactivated Suv39h1 from chromatin. To test our hypothesis, we performed a rescue experiment in MEFs derived from either WT or Suv39h1/h2 double knockout mice (Suv39h KO)^49^. We re-expressed Myc-Suv39h1 as either full length (FL), mutated in the three cysteine residues found in our MS analysis (*Suv39h1-3C* or simply, *3C*), or as a mutant with eight of the nine conserved cysteine residues (*Suv39h1-8C* or simply, *8C*) of the PRE-SET domain (Fig. 3e). As these cysteine residues are required for the structure of the PRE-SET domain, we reasoned that these mutants should have a drastic impact in the structure of the domain, in a similar way as mUb. We clearly observed that the only HMT that was able to rescue the global levels of H3K9me3 was FL (Fig. 3f). The explanation for that result became clear when we checked the localization of these proteins. Strikingly, only Suv39h1 WT relocalized in PCH foci, where it restored the H3K9me3 levels. Contrariwise, both 3C and 8C exhibited a disperse localization outside of the PCH foci, in which, accordingly, the H3K9me3 was not recovered (Fig. 3g). In agreement with these findings, in these cells only FL expression was able to recover the silencing of minor and major satellites, and of LINE-L1s (Fig. 3h).

### TNFα induces SirT6-dependent mUb of Suv39h1

**Fig. 4:**
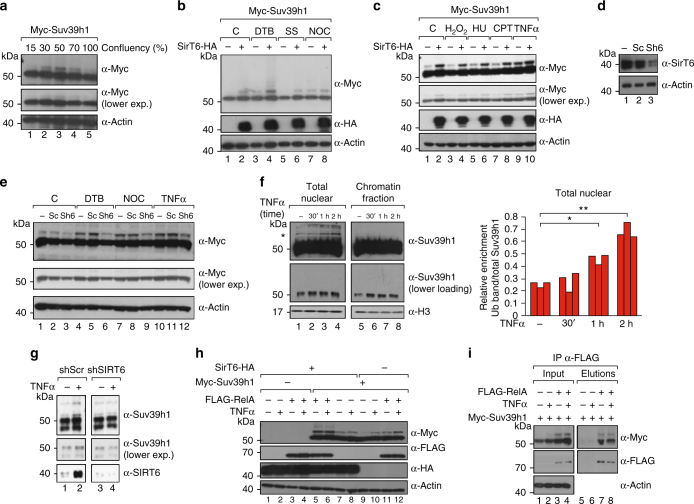
SirT6-induced monoubiquitination of Suv39h1 is induced by NF-κB pathway activation. **a** Suv39h1 levels from 293F cells transfected with Myc-Suv39h1 − / + SirT6-HA and analyzed at different degree of confluency. Cells were all plated at the same time and harvested at the indicated confluency (indicated in %). A quantification of *n* = 3 experiments is shown in Supplementary Figure 4a. **b** Western blotting of Myc-Suv39h1 − / + SirT6 expressed in 293F cells in the indicated conditions. C, control; DTB, double thymidine block; SS, serum starvation; NOC, nocodazole. A quantification of *n* = 3 experiments is shown in Supplementary Figure 4c. **c** Similar experiment as in **b**, with the indicated treatments. C, control; HU, hydroxyurea; CPT, camptothecin. FACS analysis of these treatments are included in Supplementary Figure 4b. A quantification of *n* = 3 experiments is shown in Supplementary Figure 5b. **d** SirT6 depletion by shRNA. Western blotting of endogenous SirT6 in 293F cells transfected with either scramble shRNA (Sc) or SIRT6 shRNA (Sh6). **e** Cells in **d** treated with the indicated conditions. A quantification of *n* = 3 experiments is shown in Supplementary Figure 5e. **f** Nuclear fractionation of Suv39h1 monoubiquitination induced by TNFα in 293F cells. Left panel, either total nuclear fraction or nuclear pellet digested with Benzonase (see online Methods) is shown. Right panel, quantification (*n* = 3) of the relative abundance of Suv39h1mUb vs total Suv39h1 in total nuclear fraction. (*T*-test; SEM, *p < 0.05, **p < 0.01). **g** Induction of Suv39h1mUb by TNFα in 293F cells expressing either scramble shRNA or shSIRT6. **h** Western blotting of extracts from 293F cells transfected with the indicated combinations of SirT6-HA, Myc-Suv39h1, and FLAG-RelA, and incubated in the presence or absence of TNFα before collecting the cells. **i** Co-immunoprecipitation experiments using anti-FLAG resin of extracts from 293F cells transfected with the indicated combinations of FLAG-RelA and Myc-Suv39h1 and in presence or absence of TNFα

We next sought to determine the functional implications of cysteine mUb in Suv39h1. First, we observed that it appeared to be directly related to proliferation, as its cellular levels peaked between 30 and 70% cell confluence and dropped to nil at 100% confluence (i.e., when the cells had stopped dividing; Fig. 4a and Supplementary Figure 4a). To explore this relationship, we stopped cells at various phases of the cell cycle—G_1_/early S-phase (via double thymidine block), G_0_ (by serum starvation), and in early mitosis (with nocodazole)—and then evaluated the capacity of SirT6 to induce mUb of Suv39h1 relative to control (untreated) cells. Interestingly, even without SirT6 overexpression cells halted in G_1_/S-phase or in early mitosis exhibited much higher levels of Suv39h1mUb than control cells: the levels in the S-phase cells were the highest, whereas those in the G_0_-phase cells were lower than in the control (Fig. 4b and Supplementary Figure 4b, c). Interestingly, this increased levels of the modification in S-phase correlates with the observation that SirT6 binds to many of its targets during S-phase^16^. Overall, these data corroborated a role for Suv39h1mUb in cell-cycle progression.

We then endeavored to ascertain whether any stress stimuli could induce specifically the modification. To this end, we irradiated cells to damage their DNA, and then compared their levels of monoubiquitinated Suv39h1 to those in control cells (Supplementary Figure 5a). However, we did not observe any significant effects. This was a very surprising result, given the known role of SirT6 in DNA repair^23,25^. We then tested for the effects of other forms of stress. Interestingly, oxidative stress (H_2_O_2_) and replicative stress (hydroxyurea or camptothecin) did not affect the modification, but the treatment with the cytokine TNFα, an activator of the NF-κB pathway, induced increased levels of Suv39h1mUb (Fig. 4c and Supplementary Figure 5b, c). Interestingly, TNFα treatment did not seem to alter the localization of Suv39h1 in the foci of pericentric heterochromatin (Supplementary Figure 5d). Furthermore, silencing of SirT6 by shRNA (Sh6) considerably decreased the levels of the modification upon TNFα induction, whereas treatment with scramble shRNA (Sc) did not (Fig. 4d, e and Supplementary Figure 5e). Interestingly, the effect of SirT6 downregulation on Suv39h1mUb was very mild, which suggested that the role of SirT6 on Suv39h1 is relevant in the context of specific stimuli such as TNFα activation. Together, these results suggested a role for SirT6 in inducing mUb in the context of the NF-κB pathway. Confirming the biological significance of our findings, TNFα treatment also induced the modification in endogenous Suv39h1 (Fig. 4f). As shown for Myc-tagged Suv39h1, modified endogenous Suv39h1 induced by TNFα was excluded from chromatin insoluble fraction (Fig. 4f) and abrogated upon SIRT6 downregulation by shRNA (Fig. 4g). In contrast to the partial effect on overexpressed Myc-Suv39h1, SirT6 downregulation completely abrogated the ability of TNFα to induce endogenous Suv39h1mUb (Fig. 4g, lane 2 vs 4), which strongly suggested that SirT6 is the main mediator of TNFα-induced Suv39h1mUb.

Considering the above findings in light of the aforementioned link between SirT6 and NF-κB, we reasoned that Suv39h1mUb by SirT6 might be involved in the NF-κB pathway. In line with this premise, we found that overexpression of RelA (the p65 NF-κB member) induced Suv39h1mUb equally in both Myc-tagged (Fig. 4h) or untagged Suv39h1 (Supplementary Figure 5f). Interestingly, RelA had a maximal effect on co-overexpression with SirT6 (Fig. 4h), which suggested that an important part of the effect of RelA on this mUb is mediated by SirT6. Moreover, the ability of TNFα to increase the modification was abrogated when RelA and SirT6 were both overexpressed (Fig. 4h), but was conserved when RelA was overexpressed and SirT6 levels were normal (Fig. 4h). These observations suggested that TNFα acts through both SIRT6 and RelA. Lastly, immunoprecipitation experiments revealed that RelA interacts directly with Suv39h1 (Fig. 4i) in the absence or presence of TNFα, further corroborating a link between the modification and NF-κB.

### The E3-ubiquitin ligase SKP2 is involved in Suv39h1mUb

**Fig. 5:**
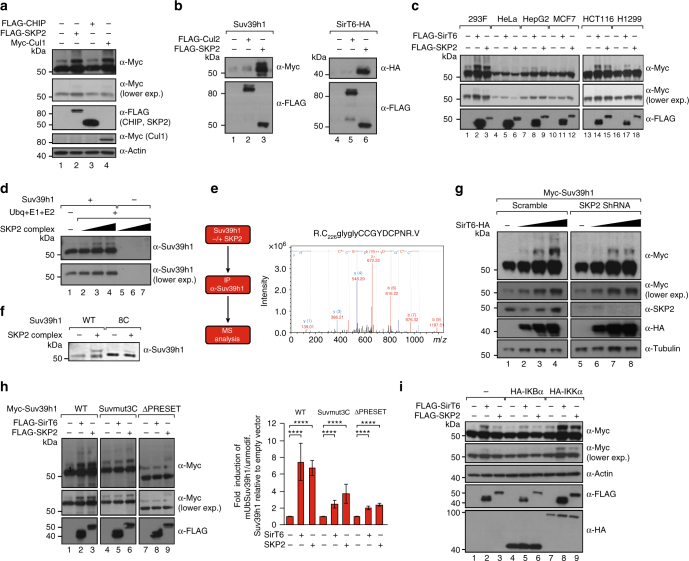
The E3-ubiquitin ligase SKP2 is involved in the SirT6-induced ubiquitination of Suv39h1. **a** Western blotting of Myc, FLAG, and β-actin from 293F extracts transfected with Myc-Suv39h1 in the presence of empty vector, FLAG-ChIP, FLAG-SKP2, or Myc-Cul1. A quantification of *n* = 3 experiments is shown in Supplementary Figure 6a. **b** FLAG immunoprecipitation of extracts from 293F cells transfected with either FLAG-SKP2 or FLAG-Cul2 together with either Myc-Suv39h1 (left panel, lanes 1–3) or SirT6-HA (right panel, lanes 4-6). **c** Analysis of Myc-Suv39h1 cotransfected − / + either FLAG-SirT6 or FLAG-SKP2 in the indicated cell lines. A quantification of *n* = 3 experiments is shown in Supplementary Figure 6e. **d** In vitro ubiquitination assay of Suv39h1 by SKP2-SCF complex. A titration of purified SCF-SKP2 complex was incubated with Suv39h1 in presence or absence of E1, E2 (Ubch3 and Ubh5c) and ubiquitin as indicated (see Online Methods). **e** Mass spectrometry analysis of Myc-Suv39h1 purified from cells co-expressing either empty vector or FLAG-SKP2. Left, scheme of the performed procedure. Right, K.AGQPIYECNSR.C peptide MS/MS spectra corresponding to the ubiquitinated precursor *m*/*z* 681,2780, containing cysteine 222 with the Gly-Gly modification. Additional data are shown in Supplementary Figure 4d and Supplementary Data Set 1. **f** Similar experiment as in **d** using Suv39h1 full-length (Wt) or Suv39h1-8C mutant as substrate. **g** Analysis of SirT6-induced monoubiquitination of Suv39h1 in 293F cells expressing scramble or SKP2-specific shRNA. A lower exposition of Suv39h1 levels is shown. A quantification of *n* = 3 experiments is shown in Supplementary Figure 6g. **h** Analysis of the effect of FLAG-SirT6 or FLAG-SKP2 overexpression in Myc-Suv39h1 WT, Suv39h1-3C (Suvmut3C) mutant (see Fig. 3e) or a deletion mutant lacking the PRE-SET domain (aa 179–240, Fig. 3c). Right panel, quantification of *n* = 5 experiments of relative abundance of Suv39h1mUb vs total Myc-Suv39h1. (*T*-test; SEM, ****p < 0.001). **i** Similar experiment as in **h**, but this time determining the impact of FLAG-SirT6 or FLAG-SKP2 overexpression in WT Myc-Suv39h1 upon co-transfection of empty vector, IκBα-HA or IKKαα-HA. A quantification of *n* = 3 experiments is shown in Supplementary Figure 6h

The SirT6 interactome has been extensively studied, leading to identification of numerous interacting partners^50,51^. Seeking to identify the E3-ubiquitin ligase involved in Suv39h1mUb, we closely examined the two E3 that have been described as SirT6-interacting partners: chromatin immunoprecipitation (ChIP) and the SKP, Cullin, F-box containing complex (SCF)-related SKP2^52,53^. We first tested each one for the ability to induce Suv39h1mUb: only SKP2 exhibited this activity. Consistently, overexpression of scaffold factor Cul1, a partner of SKP2 in the SCF complex, also induced mUb (Fig. 5a and Supplementary Figure 6a). Other pieces of evidence strongly supported SKP2 as being the E3-ubiquitin involved in this modification. First, SKP2 specifically interacts with Suv39h1, SirT6 and RelA (Fig. 5b and Supplementary Figure 5b, c). The interaction between SirT6 and SKP2 did not require SirT6 catalytic activity as the SirT6 catalytic-inactive point mutant H133Y bound to SKP2 with a similar efficiency as the WT protein (Supplementary Figure 6c). We also detected interaction of SKP2 and SIRT6 with members of the IKK complex (Supplementary Figure 6d), suggesting a functional link between all three factors in NF-κB pathway activation. Second, the ability of SKP2 to induce the mark was restricted to the same cell types in which SirT6 induced it in Supplementary Figure 2b (Fig. 5c and Supplementary Figure 6e). Third, SKP2 catalyzed Suv39h1 in vitro mUb (Fig. 5d). Accordingly, the in vitro Suv39h1mUb by SKP2 was drastically reduced in Suv39h1-8C mutant (Fig. 5f). Furthermore, MS analysis of the modification induced by SKP2 overexpression also identified cysteine mUb in the PRE-SET domain of Suv39h1 (Fig. 5e and Supplementary Figure 6f). Fourth, shRNA-induced decrease of SKP2 levels in 293F cells^54^ dramatically decreased the ability of SirT6 to induce Suv39h1mUb (Fig. 5g and Supplementary Figure 6g). Fifth, supporting the link between SKP2 and the PRE-SET domain, the ability of SirT6 or SKP2 to induce Suv39h1mUb was drastically reduced in the mutant Suv39h1-3C (Suvmut3C) as well as in a ΔPRE-SET mutant of Suv39h1, which lacks the PRE-SET domain (Fig. 5h). Lastly, overexpression of IκBα, a general repressor of the NF-κB pathway, inhibited the ability of both to induce the modification, while overexpression of the activator IKKα increased it (Fig. 5i and Supplementary Figure 6h). These last findings confirmed that the Suv39h1mUb induced by SirT6 or SKP2 is somehow linked to said pathway.

### SirT6 stabilizes SKP2 through deacetylation of the NLS

**Fig. 6:**
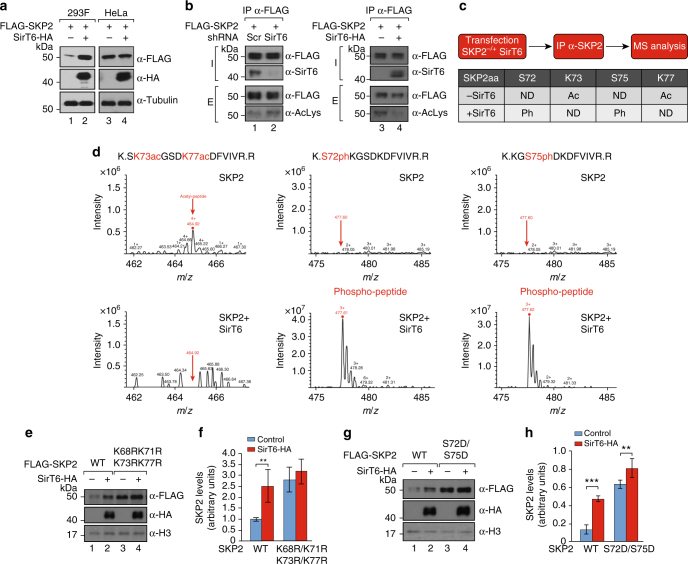
SirT6 regulates nuclear levels of SKP2 through deacetylation. **a** Analysis of the effect of SirT6 on the SKP2 levels. 293F and HeLa cells were co-transfected with the indicated combinations of FLAG-SKP2 and SirT6-HA. Tubulin was included as loading control. **b** Levels of acetylation in FLAG-SKP2 purified from 293F cells downregulated in SirT6 (shScramble vs shSirT6, left) or overexpressing SirT6-HA in 293F cells expressing shRNA SirT6 (right). SKP2 was purified with FLAG resin and analyzed by western-blot with anti-acetyl-lysine antibodies. **c** Posttranslational modifications in SKP2 in the presence or absence of SirT6. Upper panel: Summary of the procedure. Lower panel: Summary of the identified PTMs in SKP2 in the indicated conditions. Additional data is shown in Supplementary Data Set 2. **d** Left, ESI-MS spectrum of acetylated peptide (aa 72–83, ^72^K.SK73acGSDK77acDFVIVR.R.^83^-C) from SKP2 − / + SirT6 of a representive experiment of two replicas. Signal was detected at *m*/*z* 464.92 (charge state 4). Middle and right, ESI-MS spectrum of phosphorylated peptides (aa 72–83, ^72^K.S72phKGS75phDKDFVIVR.R.^83^-C, ^72^K.KGS75phDKDFVIVR.R.^83^-C) from SKP2 − / + SirT6. Signal appeared at *m*/*z* 477.61 and 477.61, respectively). **e** Analysis of the ability of SirT6-HA to upregulate the levels of nuclear FLAG-tagged SKP2, as either WT or tetramutated in K68R/K71R/K73R/K77R. Western blotting of nuclear extracts from 293F cells previously transfected with the indicated constructs. **f** Quantification of *n* = 5 experiments as the one showed in **e**. SKP2 levels were normalized with histone H3. All the values were represented relative to the normalized levels of WT SKP2 in the absence of SirT6 (*T*-test; s.e.m., **:p < 0.01). **g** Same experiment as in **c**, **d**, but testing the levels of nuclear SKP2 double mutant S72D/S75D instead of the tetramutant. **h** Quantification of *n* = 6 experiments as in **g** and represented as in **f** (*T*-test; SEM, *p < 0.05, ***p < 0.005)

We next aimed to define the mechanism through which SirT6 regulates mUb of Suv39h1. We noted that increased levels of SirT6 correlated with increased levels of SKP2 in 293F cells, but not in HeLa cells (Fig. 6a and Supplementary Figure 7a). Moreover, HA-tagged ubiquitin was incorporated into Suv39h1 upon overexpression of either SirT6 or SKP2 in 293F but not in HeLa cells (Supplementary Figure 7a). These observations suggested a direct link between regulation of SKP2 stability by SirT6, and the ability of SKP2 to monoubiquitinate Suv39h1. As shown before (Fig. 2e), SirT6 requires its deacetylase activity for this function. As we were unable to identify Suv39h1 as a SirT6 substrate, we tested whether SKP2 was targeted by SirT6. We first observed that SIRT6 downregulation overexpression induced an increase in acetylation levels of SKP2 (monitored by anti acetyl-lysine antibodies), whereas SIRT6 overexpression in SIRT6-downregulated cells induced the opposite effect (Fig. 6b). MS analysis of SKP2 purified from cells expressing SKP2 alone or together with SirT6, we did identify two lysine residues (K73 and K77) that were deacetylated in the presence of SirT6 (Fig. 6c, d and Supplementary Data Set 2). Strikingly, these two residues are located in the nuclear localization signal of SKP2. Previous studies showed that acetylation of these two residues, as well as nearby K68 and K71, were involved not only in the localization of SKP2, but also in stabilizing SKP2 protein^52^ through inhibition of S72 and S75 phophorylation in SKP2^55,56^. Further supporting a role for SirT6 in SKP2 stability (through lysine deacetylation), the presence of SirT6 correlated with a significant decrease in the nuclear acetylation levels of these two lysines and with a significant increase in the phosphorylation levels of the two close serine residues S72 and S75 (Fig. 6c, d and Supplementary Data Set 2). Phosphorylation of S72 by Akt triggers subsequent phosphorylation of S75 by Casein Kinase I. In turn, when S72 and S75 are phosphorylated, the binding of APC-Cdh1 E3 ubiquitin-ligase is inhibited and consequently, subsequent degradation of SKP2 is prevented^57,58^.

To confirm the involvement of SirT6 in this pathway, we generated mutants containing different combinations of lysine-to-arginine mutations (as mimics of deacetylated lysine) at the four conserved lysine residues (K68, K71, K73, and K77) in the NLS of SKP2. In agreement with that, mutation of these four residues resulted in elevated nuclear levels of SKP2 that were not further impacted by overexpression of SirT6. Interestingly, we only observed this effect when all four lysine residues were mutated (Fig. 6e, f and Supplementary Figure 7b), indicating that K68 and K71 are also SirT6 targets. Accordingly, we obtained a similar result when we mutated S72 and S75 to aspartic acid (as mimics of phosphorylated serine) (Fig. 6g, h). Altogether, these findings reflect the importance of stabilization of nuclear SKP2 via deacetylation by SirT6.

### IκBα expression is regulated by SirT6, Suv39h1 and SKP2

**Fig. 7:**
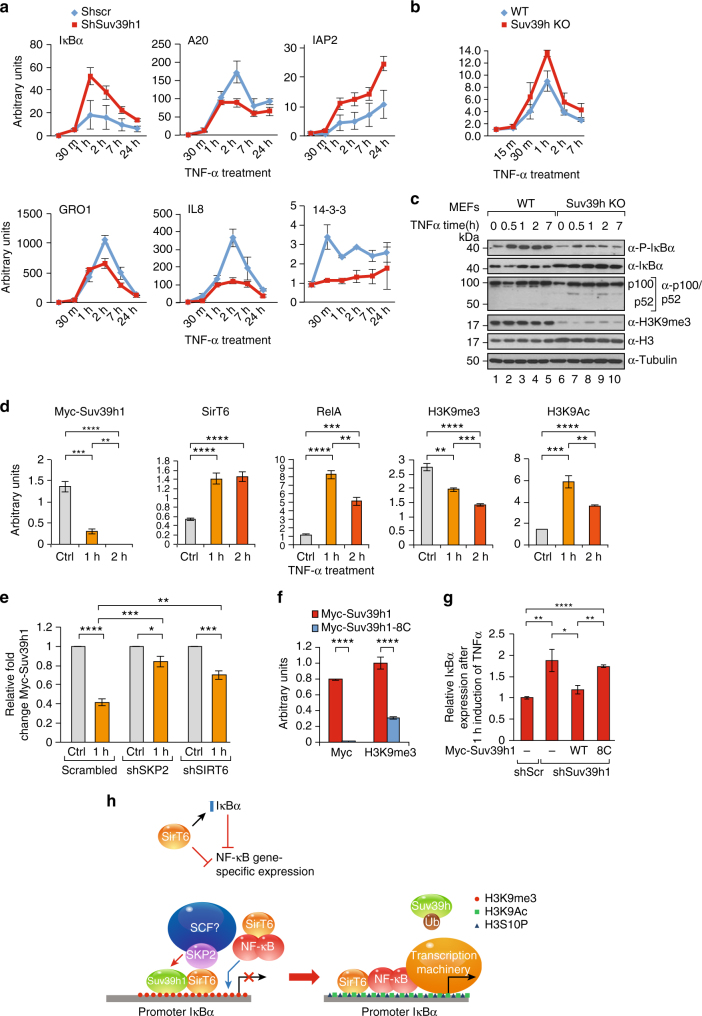
Suv39h1 and SirT6 regulate *IkBα* expression upon NF-κB pathway activation. **a** Quantitative RT-PCR analysis (*n* = 3, SD) of mRNA from 293F cells transfected with scramble shRNA or shSuv39h1 and treated with TNFα. Cells were harvested at the indicated times of TNFα treatment. A representative group of early genes activated by NF-κB pathway upon TNFα induction were analyzed. **b** Same experiment as in **a** of *IκBα* expression upon TNFα induction in WT and Suv39h KO MEFs (*n* = 3, s.d). **c** Western blotting analysis of the levels of IκBα,phospho-IκBα (P-IκBα), p100, p52 and H3K92m3 in WT and Suv39h KO MEFs treated with TNFα the indicated times. Tubulin and histone H3 were used as loading controls. **d** ChIP analysis of the indicated factors in the IκBα promoter of 293F cells in non-induced conditions (ctrol) or upon treatment of TNFα for 1 and 2 h. Given the lack of a reliable Suv39h1 antibody, in this case we transfected the cells with Myc-Suv39h1 and performed the ChIP analysis with α-myc antibody. The genomic *IκBα* promoter was quantified with qRT-PCR(representative of *n* = 3; *T*-test; SEM, **p < 0.01, ***p < 0.005, ****p < 0.001). **e** ChIP analysis of *IκBα* promoter of Suv39h1 in 293F cells expressing shRNA scramble, shSirT6, or shSKP2 and analyzed as in **d** (*n* = 5; *T*-test; SEM, *p < 0.05, **p < 0.01, ***p < 0.005, ****p < 0.001). **f** ChIP analysis of *IκBα* promoter with the indicated antibodies of 293F cells transfected with Myc-Suv39h1 either WT or the 8C mutant described in Fig. 3 upon 1 h of TNFα induction(representative of *n* = 3; *T*-test; SEM, ****p < 0.001). **g** Rescue experiment of human 293F expressing shScramble or shSuv39h1 with mouse Suv39h1 WT or 8C. Relative expression levels of IkBα upon 1 h of TNFα induction relative to the expression levels in shScramble cells. ((*n* = 5; *T*-test; SEM, *p < 0.05; **p < 0.01; ****p < 0.001). **h** Model of the novel mechanism proposed for the regulation of *IκBα* expression by the interplay between Suv39h1, SirT6, and SKP2

We next aimed to define the functional consequences of the mechanism that we had identified—namely, to ascertain the contribution of Suv39h1 to the NF-κB pathway. Thus, we studied the expression of various genes targeted by NF-κB in response to TNFα treatment upon loss of Suv39h1. We tracked gene expression at several time points following the treatment: 0 h, 30 min, 1 h, 2 h, 7 h, and 24 h (Fig. 7a). Considering that Suv39h1 had previously been identified in the promoters of at least one gene induced by TNFα^38^, we were expecting that an shRNA-induced decrease in Suv39h1 mRNA levels in more than 60% (Supplementary Figure 7a) would result, if anything, in an upregulation of a set of genes. However, upon loss of Suv39h1 and following TNFα treatment, many of the genes tested exhibited attenuated expression (Fig. 7a), suggesting that Suv39h1 is required in these cells to enable full activation of the pathway. Interestingly, for the vast majority of these genes, expression peaked at *ca*. 2 h of TNFα treatment. In contrast, a few genes were upregulated under these conditions, including the general repressor *IκBα*, an early-activated gene whose expression peaked at *ca*. 1 h of TNFα treatment. Supporting the observation, the response of *IκBα* expression to TNFα treatment in MEFs cells deficient for Suv39h1/2 (Suv39hKO) showed an identical profile (Fig. 7b). Interestingly, loss of SirT6 by shRNA in a similar experiment as in Fig. 7a showed the opposite effect on *IκBα* expression upon TNFα treatment, suggesting an antagonism between SirT6 and Suv39h1 in the regulation of the IκBα gene expression (Supplementary Figure 7b). This observation would fit with our previous results indicating Suv39h1 chromatin eviction induced by mUb (Figs. 2h-j, 3g, and 4f). Based on our results, we postulated that Suv39h1 might be required for full activation of the NF-κB pathway, because it would be important for silencing of *IκBα* expression, thereby globally regulating the entire pathway and affecting numerous target genes. In this scenario, the signal in these genes would be attenuated via upregulation of the repressor IκBα, which in turn would induce less nuclear RelA and less gene expression. To confirm our hypothesis, we next analyzed the status of the NF-κB pathway in Suv39h1/2-defficient MEFs (Suv39hKO) at the protein level. Strikingly, these cells not only harbored significantly higher levels of IκBα protein, but also showed lower levels of pathway activation, as measured by phospho-IκBα (Fig. 7c). As expected, the levels of p100, which is a target of canonical NF-κB, were slightly increased in WT cells and modulated by TNFα treatment. Identical results were detected in an additional set of WT and Suv39hKO MEFs (Supplementary Figure 7c). However, we failed to detect consistent difference in the processing of p100 to p52 between WT and knockout MEFs in the different experiments performed, suggesting that alternative NF-κB pathway is not a directly targeted by Suv39h1/2 function.

Given the cardinal role of IκBα, we asked ourselves whether SirT6-induced regulation of Suv39h1 might occur in this context. To answer this question, we performed ChIP experiments in which we treated 293F cells with TNFα and then quantified the levels of Suv39h1, SirT6, RelA H3K9me3, and H3K9Ac in the *IκBα* promoter at 0, 1, and 2 h of treatment (Fig. 7d). Although we observed Suv39h1 in the promoter at t0 (control), it had dramatically decreased in occupancy by 1 h of treatment and had completely disappeared by 2 h. In contrast, SirT6 was present in low levels at t0, strongly increased in occupancy within 1 h, and then plateaued up through 2 h. Interestingly, RelA levels had peaked at 1 h and decreased significantly around 2 h. Consistently with these observations, the levels of H3K9me3 decreased in parallel to those of Suv39h1 in the promoter, although at 2 h the levels of H3K9me3 had not decreased below 50% of the levels at t0. This decrease was also associated with an increase in H3K9Ac levels from t0 to 1 h, although they decreased at 2 h, reflecting both the decrease in RelA and the plateau of SirT6 in the promoter. In order to confirm our hypothesis that both SirT6 and SKP2 play a role in Suv39h1 dynamics in the *IκBα* promoter, we performed ChIP assays of Suv39h1 in SirT6 or SKP2-depleted cells. As expected, we observed that shRNA-driven depletion of SirT6 or SKP2 significantly inhibited Suv39h1 eviction from *IκBα* promoter after 1 h of TNFα treatment, resulting in retention of 83.35% of initial Suv39h1 in the case of SKP2 and 70.85% in the case of SIRT6 (Fig. 7e).

To confirm the role of the cysteine residues in Suv39h1 localization in *IκBα* promoter, we next expressed Suv39h1 in WT or 8C-mutant 293F cells without using any TNFα treatment. Supporting our hypothesis, the 8C mutant was not present in the promoter, and this correlated with a concomitant dramatic decrease of H3K9me3 levels in the promoter compared to WT cells (Fig. 7f). Consistently, expression of mouse Suv39h1 WT but not 8C could rescue *IkBα* expression in human 293F cells with downregulated Suv39h1 (Fig. 7g). Altogether, our findings support our model suggesting that Suv39h1 plays a crucial role in activation of the NF-κB pathway by controlling *IκBα* expression.

## Discussion

We have identified a mechanism of action for gene expression in the context of NF-κB pathway, whereby SirT6 induces mUb of cysteine residues in the PRE-SET domain of Suv39h1. Of all the regular conditions that trigger activation of sirtuins, our findings indicate that only activation of the NF-κB pathway through TNFα treatment or RelA overexpression induces this modification in Suv39h1 (Fig. 4). Moreover, overexpression of either the pathway activator IKKα or the inhibitor *IκBα* promotes a drastic increase or decrease, respectively, in the levels of monoubiquitinated Suv39h1 (Fig. 5). This stress specificity is striking, as DNA damage signaling seems to be one of the key activation signals of SirT6^23,59^. This observation strongly suggests that Suv39h1mUb does not have a general role in Suv39h1 function and is involved in a restricted and specific function. Our results from blocking cells in G_1_/S (by double thymidine block) or early M (by Nocodazole) supports the link between this modification and the NF-κB pathway, which regulates both of these cell cycle transitions^60,61^. Based on our evidence, we propose a model whereby SirT6 controls the NF-κB pathway globally by regulating *IκBα* expression (Fig. 7h). Before TNFα treatment, Suv39h1 and SirT6 are present in the* IκBα* gene. Activation of the NF-κB pathway by TNFα activates SirT6, which in turn activates SKP2 by deacetylating it, which leads to subsequent phosphorylation of S72 and S75. These results are in contrast to other previous work that identified acetylation of K68 and K71 as promoters of SKP2 stability^52^. Interestingly, our studies suggest a more complex model, as mutation of all four lysine residues (K68, K71, K73, and K77) induces S72/S75 phosphorylation and subsequent upregulation of SKP2 levels. Considering the described effect of K68 and K71 acetylation in SKP2 localization, a possible explanation for these discrepancies may lie in the fact that our studies are dealing with a nuclear population of SKP2, which may not be regulated as cytoplasmic SKP2. Interestingly, SirT6 and Akt, responsible for S72 phosphorylation, have been shown to interact directly^62^, which may also indicate a direct effect of SirT6 on Akt activity. Our model suggests that Suv39h1 ubiquitinates by SKP2 and promotes its exclusion from chromatin, thereby enabling demethylation of H3K9 and subsequent phosphorylation at H3S10 by IKKα. Surprisingly, the levels of SirT6 and RelA do not parallel each other at 2 h of TNFα treatment, as by this time RelA enrichment in the promoter has decreased but not SirT6. This suggests that other, unknown mechanisms regulate the silencing of NF-κB by SirT6.

Interestingly, we have observed that the effect of SIRT6 on Suv39h1 is cell type specific. In our experiments, SIRT6 induced Suv39h1mUb in 293F (embryonic kidney), H1299 (lung), HCT116 (colon), and NIH3T3 (mouse multipotent cells) but not in HeLa (epithelial), MCF7 (breast), or U2OS (bone) (Fig. 5c and Supplementary Figure 2b). The reason for this specificity is unclear. A possible explanation may be NF-κB pathway itself, as it has been shown that the mechanisms driven by the pathway upon stress are very diverse depending on the stimuli involved and the cell type^63^.

mUb of proteins in lysine residues has long been known. This reversible modification can alter the activity, structure, or localization of proteins. To date, it has been reported in cell signaling, chromatin structure, transcription, endocytosis, intracellular trafficking, and even stress response^55,56^. There is extensive literature on lysine mUb in regulation of the NF-κB pathway, an activity that seems to involve several factors, including RelA and NEMO (IKKγ)^64^. Despite the dogma that ubiquitination chiefly occurs in the ε-amino group of lysine residues through an isopeptide bond, recent evidence demonstrates that this modification is far broader, as it can also affect serines, threonines, and cysteines^45,65^. In this case, the resulting linkage is not an isopeptide bond, but either a hydroxyester (for serine and threonine) or thioester (for cysteine) bond. Our knowledge on such non-canonical ubiquitination is very limited. So far, they have been reported in immunosuppression by viral E3-Ub ligases, in peroxisomal import^66,67^, and (serine and threonine only) in degradation of defective ER proteins by viral and mammalian E3-Ub ligases^45,65,68^. Interestingly, all of these cases involve polyubiquitination and subsequent degradation of the target protein, except for one: mUb of cysteines in the context of peroxisomal function in yeast^69^.

Here we have reported that SirT6 induces mUb of Suv39h1 at four cysteines (C49, C222, C226, and C232) and one serine (S29). Three of these residues are present in the PRE-SET domain, a region comprising *ca*. 75–100 residues that is located just before the catalytic SET domain. The PRE-SET domain is not general among HMTs: in fact, is only present in the members of three of the SET-containing HMT families, including the SUV39 family (which includes Suv39h1 and G9a)^70^. In this family of HMTs, the PRE-SET domain contains nine conserved cysteines (CXCX_5_CX_4_CXCXN-CX_3_CXCX_3_C), which coordinate three Zn^+2^ molecules in a triangular structure^71,72^. The role of this domain has not been fully characterized. Some authors have suggested that it has a structural role, or that it participates in protein dimerization and binding to ssDNA or RNA^73^. One major clue stems from comparison of the PRE-SET domain and the CXC domain of the component of the *Drosophila* Dosage Compensation Complex, MSL2^46^. Surprisingly, these two domains are similar: similar to the PRE-SET domain, the CXC domain contains nine cysteines that coordinate three Zn^+2^ ions (Zn_3_Cys_9_) and its recently solved structure strikingly resembles that of the former^71^. The fact that MSL2 is a DNA-binding protein required for proper targeting of the male X-chromosome in *Drosophila*^74^ strongly suggests that the PRE-SET domain is involved in establishing links between Suv39h1 and DNA upon binding of Suv39h1 to chromatin. Our results fully support this model as Suv39h1mUb is excluded from tight chromatin (Fig. 2g–i), and mutation of the three modified cysteines (Suv39h1–3C) abrogated both localization of Suv39h1, as well as its capacity to catalyze H3K9me3 in CH foci (Fig. 3e–h).

Although here we have focused on the PRE-SET domain, we show that Suv39h1 is monoubiquitinated outside this domain. This is clearly demonstrated both by our MS analysis, but also by the significant equivalent mUb levels detected in ΔPRESET and 3C (Suvmut3C) (Fig. 5h). Our demonstration that the cysteines of the PRE-SET domain are the main targets of SirT6-induced Suv39h1mUb as shRNA-driven downregulation of SirT6 abrogated partially or completely overexpressed or endogenous Suv39h1mUb, respectively (Fig. 4e, h and Supplementary Figure 5e). Nevertheless, the other identified mUb outside of the PRE-SET domain are also potentially interesting, given their location in two nearby regions or domains of Suv39h1 that are critical for binding to chromatin: serine 29 is located in the N-terminal region (1–43 residues), which is the binding site for HP1 proteins^75^; and cysteine 49 is part of the H3K9me3-binding chromodomain, which spans residues 44–88. We hypothesize that mUb of S29 or C49 might be equivalent to abrogating the ability of Suv39h1 to bind to chromatin. Our hypothesis is supported by the previous finding that a Suv39h1 deletion mutant that lacks the first 89 N-terminal residues completely lost the ability to bind to chromatin in metaphasic chromosomes^75^.

Here we have also identified SKP2 as the main E3-ubiquitin ligase that catalyzes mUb of cysteines in Suv39h1. SKP2, a component of the SCF multi-protein E3-ubiquitin ligase complexes, has always been linked to polyubiquitination—and therefore, to degradation—of crucial regulators of the cell cycle, apoptosis, etc. Although our data suggests that SKP2 is the main E3 activity involved in SirT6-dependent Suv39h1mUb, this may not be the only one as SKP2 downregulation did not completely abrogate the modification (Fig. 5g). In fact, we cannot exclude that this may be result of another E3 ligase activity or that this remaining modification may involve ubiquitin-like proteins instead of ubiquitin. One open question is whether the protein partners and the mechanism of action of SKP2 in this context are the same SCF components involved in polyubiquitination of proteins. Supporting that this may be the case, overexpression of a partner of SKP2 in the SCF complex, the scaffold protein Cul1, also induced the modification in contrast to Cul2, a SCF-unrelated close relative (Figs. 5a, b). Interestingly, SCF-dependent protein polyubiquitination requires specific phosphorylation of the target^76,77^. However, we have been unable to identify any Suv39h1 phosphorylation related to SirT6-dependent mUb.

In summary, we describe a new mechanism in chromatin regulation based on monoubiquitination of cysteines . We hypothesize that this modification, which regulates access of Suv39h1 to chromatin, is reversible and represents a fast, efficient, and dynamic way to signal responses to different physiological stimuli. Similar mechanisms have been proposed for canonical (lysine) mUb in gene expression. The best example is the regulation of SMAD transcription factors under transforming growth factor-β (TGF-β) activation. TGF-β-dependent SMAD4 lysine mUb by TIF1γ/TRIM33 induces SMAD4 dissociation from the SMAD complex bound in the gene. Similarly, SMAD3 mUb inhibits binding to DNA and to the rest of SMAD complex, whereas its deubiquitination by USP15 has a reverse effect^78,79^.

Several issues remain to be resolved in future studies: for instance, whether mUb of cysteines is also present in other enzymes, involves other E3-ubiquitin ligases or associated machinery, or participates in other nuclear events. Our work opens a new path in the study of nuclear functions and suggests a much more complex regulatory landscape than that previously anticipated.

## Methods

### Antibodies and western blottings

The antibodies used were α-HA (Sigma-Aldrich H6908; WB 1 : 1,000), α-FLAG (Sigma-Aldrich F7425, WB 1 : 1,000 ChIP 5 μg), α-myc (Cell Signaling 2276 S, WB 1 : 5,000 immunofluorescence (IF) 1:150 ChIP 5 μg), α-H3K9me3 (Millipore 07–442, WB 1 : 1,000; Abcam ab8898, IF 1 : 150 ChIP 5 μg), α-H3K9ac (Cell Signaling ab1191, ChIP 5 μg), α-actin (Sigma-Aldrich A1978, WB 1 : 5,000), α-tubulin (Sigma-Aldrich T6199, WB 1 : 20,000), α-NFkB p65 (Santa Cruz Biotechnology sc-372-x, WB 1 : 1,000), α-SirT6 (AbCam ab62739, WB 1 : 1,000; ChIP 5μg), α-H3 (Cell Signaling 9715 S, WB 1 : 2,000), α-acetyl-lysine (Cell Signaling 9814 S, WB 1 : 1,000), α-suv39h1(Millipore 07–550, WB 1 : 1,000), α−SKP2 (Thermo Fisher Scientific 32–3300, WB 1 : 1,000), α-IκBα (Santa Cruz sc-371, 1 : 1,000), α-P-IκBα (Cell Signaling 9246 S, WB 1 : 1,000), and α-p100/p52 (Millipore 05-361, WB 1 : 1,000).

WBs were performed as described elsewhere. Images of original WBs included in the main figures are included in Supplementary Figure 9.

### Cells and treatments

HEK293F, HeLa, H1299, HCT116, NIH3T3, and U2OS (ATCC) were grown in DMEM (Life Technologies) supplemented by 10% fetal bovine serum (Life Technologies). MEFs (Suv39DN, Jenuwein group; *sirt1*^−/−^, Mostoslavsky group; *sirt6*^−/−^ Bober group) were supplemented with 15% fetal bovine serum. Cells were routinely tested for mycoplasm infection with PCR-detection kit (BIOTOOLS-BIOT. & MED.L.). All transfections, except in the case of MEFs, were carried out as previously described^15^ using the indicated plasmids. Cells were collected after 48–72 h for analysis. MEF cells were infected by retrovirus produced with Platinum packaging cells (Cell Biolabs) using the pMSCV vectors (Clontech).

Cells were arrested at G_1_/S phase with double thymidine block. Cells were plated at 60% of confluency and cultured for 24 h before adding 4 mM thymidine (Sigma-Aldrich) to the media. After 12 h of incubation with 4 mM thymidine, the media was removed and replaced by fresh media and cultured for 12 h. At that moment, 4 mM thymidine was added again to the media and cultured for 12 h before collecting. In the case of cells arrested at early mitosis, cells were plated and cultured for 24 h and incubated 24 h more with media containing 4 mM thymidine. The media was removed and replaced by fresh media. After 3 h, fresh media containing 250 ng ml^−1^ nocodazole (Sigma-Aldrich). The cells were collected 12 h later.

Cells under different forms of stress were transfected with the corresponding plasmids, cultured for 48 h, and treated with the following conditions before collecting: 5 mM of H_2_O_2_ (MERCK) for 2 h, 2 mM hidroxyurea (Sigma-Aldrich) for 4 h, 1 μM campthotecin (Sigma-Aldrich) for 1 h and 20 ng ml^−^^1^ of TNFα (Peprotech) for the indicated times. Cells were irradiated 48 h after transfection with 10 Gy and collected at the indicated times.

### Protein extraction and immunoprecipitation

Proteins were extracted according to the Dignam protocol^80^: first extraction for the cytoplasmic soluble fraction was made with Buffer A (10 mM Tris pH 7.8; 10 mM KCl; 1.5 mM MgCl_2_) and second extraction for the nuclear soluble fraction was made with Buffer C (10 mM Tris pH 7.8; 0.42 M NaCl; 1.5 mM MgCl_2_; 0.2 mM EDTA; 25% glycerol). In order to separate soluble and chromatin insoluble proteins, soluble proteins were first extracted with RIPA buffer (50 mM Tris-HCl pH 7.8; 150 mM NaCl; 0.5% Deoxycholic acid; 0.1% SDS; 1% NP40) and the insoluble chromatin pellet was digested with Benzonase (Sigma-Aldrich). Histone extraction was performed by acid extraction method^15^.

Recombinant GST proteins were expressed in BL21 *Escherichia coli* strain and resuspended in NETN buffer (20 mM Tris pH 7.8, 100 mM NaCl, 1 mM EDTA, and 0.5% NP40) with 0.2 and 2% sarcosyl. The proteins were purified using Glutathione sepharose beads (GE Healthcare). Beads were washed with NETN buffer (20 mM Tris pH 7.8, 100 mM NaCl, 1 mM EDTA, and 0.5% NP40) and TST buffer (50 mM Tris pH 7.8, 150 mM NaCl, and 0.1% Triton), and eluted with reduced gluthatione (Sigma-Aldrich).

For immunoprecipitation experiments, cell extracts were incubated with either α-FLAG, α-HA resin (Sigma-Aldrich), or α-Myc tag antibody (Cell Signaling) crosslinked to proteinG-Agarose resin (MERCK), overnight. Beads were washed three times with BC100 buffer (10 mM Tris pH 7.8, 0.5 mM EDTA, 0.1 mM phenylmethylsulfonyl fluoride, 0.1 mM dithiothreitol (DTT), 10% glycerol, 100 mM KCl) and five times with BC500 buffer (500 mM KCl). Then, proteins were eluted with 0.2 M Glycine pH 2 or by incubation with the corresponding competing peptides. Incubation of nuclear extracts containing myc-Suv39h1 with 100 mM NaoH was performed as described^65^

### Immunofluorescence

For IF experiments, cells were transfected and, after 24 h, were replated on coverslips and then incubated for another 24 h. Cells were fixed in 2% paraformaldehyde for 10 min at room temperature and permeabilized for 10 min with 0.1% sodium azide phosphate-buffered saline (PBS), 0.1% Triton-X, and 5% bovine serum albumin (BSA). Primary and secondary antibodies were diluted in 0.1% sodium azide PBS, 0.2% Triton-X, and 0.5-1% BSA. As secondary antibodies, anti-rabbit Alexa Flour 488 and anti-mouse Alexa Flour 568, from Molecular Probes, were used. Cells were counterstained with DAPI (4',6-diamidino-2-phenylindole; Sigma) and slides were mounted on Vectashield (Vector Laboratories). Labeled cells were imaged using a Zeiss LSM510 Meta Confocal Laser Scanning Microscope.

### In vitro enzymatics assays

The HMT in vitro assay was performed as described^13,15^ with purified proteins using HeLa purified core histones or GST-fused mutants of N-terminal histone H3 tails. The signal was enhanced by EN3HANCE spray (Perkin-Elmer) and the loading was controlled by Coomassie-Blue Staining (Sigma-Aldrich).

For the in vitro ubiquitination assay, myc-Suv39h1 WT and Suv39h1-8C purified with anti-Myc Agarose was incubated for 2 h at 30 °C in reaction buffer (20 mM Hepes-HCl pH 7.4, 10 mM MgCl_2_, 2 mM DTT, and 2.5 mM ATP) with 0.5 μg E1, 1 μg E2 (Ubch13 and Ubch5c), and 60 μM ubiquitin (Sigma-Aldrich) in the presence or absence of increasing amounts of the components of SKP2-SCF complex (Skp2, Cul1, Skp1, and Roc1). The SCF complex was previously purified with anti FLAG-agarose from cells transfected simultaneously with FLAG-SKP2, Myc-Cul1, HA-Skp1, and Myc-Roc1, by FLAG-peptide specific elution. Reactions were stopped with protein loading buffer, run in SD-polyacrylamide gel electrophoresis (PAGE) gel and the presence of Suv39h1 monoubiquitinated band was analyzed by WB.

### Liquid chromatography-MS/MS for Post-translational modification (PTM) identification

Proteins from SDS-PAGE gel bands were excised and subsequently digested with trypsin. The resulting peptides were separated by reverse-phase liquid chromatography using a nano-capillary analytical C18 column and then electrosprayed into an ion-trap mass spectrometer (Amazon ETD Ion Trap (Bruker Daltonics) and LTQ Velos-Orbitrap (ThermoScientific)). Peptide masses were analyzed at full scan MS and then at MS/MS fragmentation for the most intense peaks. Data were analyzed using the Mascot search engine and the SwissProt database. Detailed information is provided in the Supplementary Data Sets 1 and 2.

### ChIP assays

Cells were crosslinked with 1% paraformaldehyde and the reaction was stopped with 125 mM Glycine. The cells were then lysed with lysis buffer (50 mM Tris pH 7.8, 10 mM EDTA, and 1% SDS) and the chromatin was sonicated with a Bioruptor (Diagenode) until a range of 300 or 1,000 bp was reached. Samples were diluted in dilution buffer (1% Triton, 2 mM EDTA, 150 mM NaCl, 20 mM Tris pH 7.8) at least six times and then pre-cleared with Protein-G Magnetic beads (MERCK) that had been pre-incubated for at least 6 h (in rotation with 5% BSA and 1 mg ml^−1^ salmon sperm DNA). The pre-cleared samples were incubated with the indicated antibodies for at least 6 h and then incubated with Magnetic Protein G beads overnight. The beads were washed with TSE I buffer (150 mM NaCl, 0.1% SDS, 1% Triton, 2 mM EDTA, 20 mM Tris pH 7.8), TSE II buffer (500 mM NaCl, 0.1% SDS, 1% Triton, 2 mM EDTA, 20 mM Tris pH 7.8), Buffer III (0.25 M LiCl, 1% NP−40, 1% deoxycholate, 1 mM EDTA, 10 mM Tris pH7.8) and PBS 1 ×. The beads were eluted with 0.1 M NaHCO_3_ and 1% SDS with agitation. The crosslinking was reverted at 65 °C overnight and the DNA was purified. Quantitative reverse transcriptase PCR (RT-PCR) was performed using the hNFKBIA promoter primer (IκBα promoter).

### mRNA extraction, RT-PCR, and quantitative PCR

mRNA was extracted from cells using Trizol (Life Technologies) following the manufacturer’s protocol. Retrotranscriptase PCR was performed with this mRNA using Transcriptor Reverse Transcriptase (ROCHE). Quantitave PCR was performed with Sybr green Master Mix of Applied Biosystems and the primers described in Supplementary Table 2 were used. The results were normalized using primers for EEF2, HPRT1, and NCL for human samples and EEF2, HPRT1, and RPL38 for mouse samples.

### Statistical analysis

Statistical analysis was performed using bivariant *T*-test analysis. All analysis were performed with *n* ≥ 5 unless stated otherwise in the corresponding figure legends. The data analyzed fit with a normal distribution and showed no significant variance differences among sets of data according to a variance F-test analysis. The graphs represent mean values and include SE (SEM) unless stated otherwise. The *p*-values of each analysis are indicated in figure legends.

### Data availability

The data that support the findings of this study are available from the corresponding author upon request.

## Electronic supplementary material

Supplementary information

Descriptions of Additional Supplementary Files

Supplementary Dataset 1

Supplementary Dataset 2
