# Supplementary material for: SIRT6-dependent cysteine monoubiquitination in the PRE-SET domain of Suv39h1 regulates the NF-κB pathway
Source: Nat Commun. 2018 Jan 9;9:101. doi: 10.1038/s41467-017-02586-x (PMC5760577; doi:10.1038/s41467-017-02586-x)
Supplement: Supplementary file 1 — Supplementary information [file 41467_2017_2586_MOESM1_ESM.pdf]

## SUPPLEMENTAL FIGURE LEGENDS

***Santos-Barriopedro et al.***

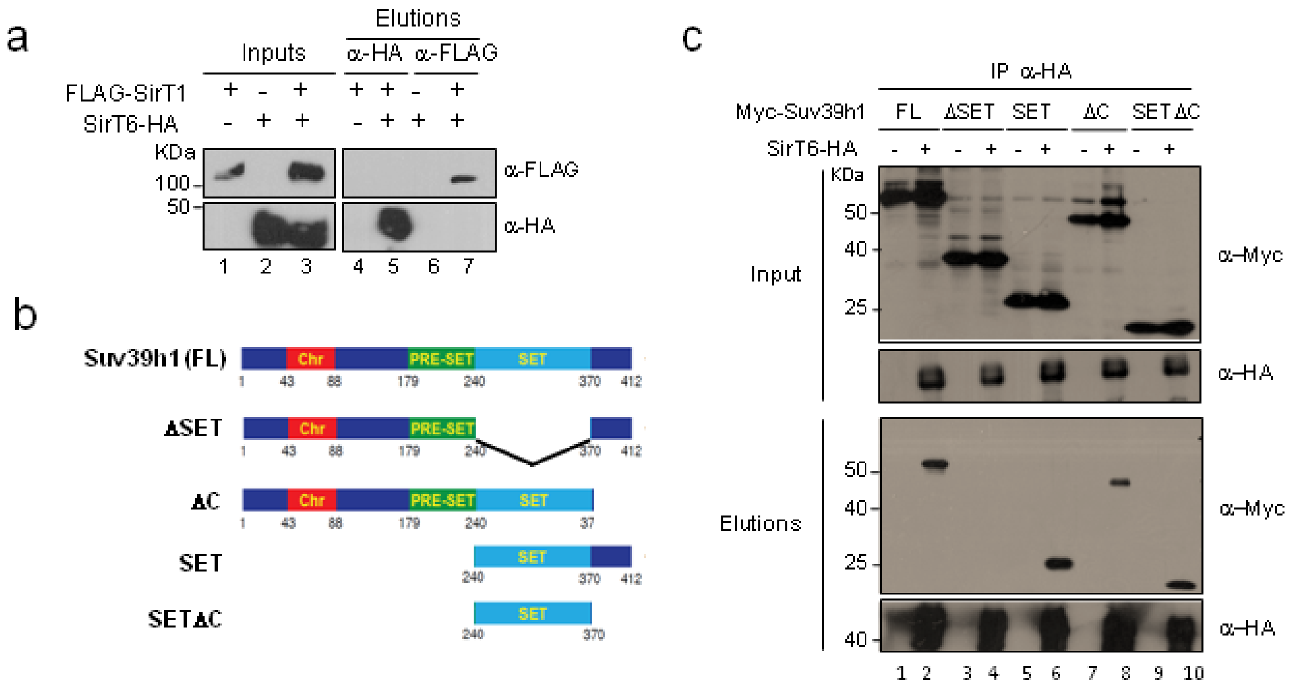


**Supplementary Fig.1**. **SirT6 interacts directly with Suv39h1 through its C-terminal catalytic SET domain. (a)** Immunoprecipitation of FLAG-SirT1 and SirT6-HA using either α-HA or α-FLAG as indicated. **(b)** Schematic representation of the Suv39h1 constructs used in (c). **(c)** Immunoprecipitation of SirT6-HA with Myc-Suv39h1 full-length (FL), ΔSET, SET, ΔC or SETΔC shown in (b). Inputs and elutions are shown.

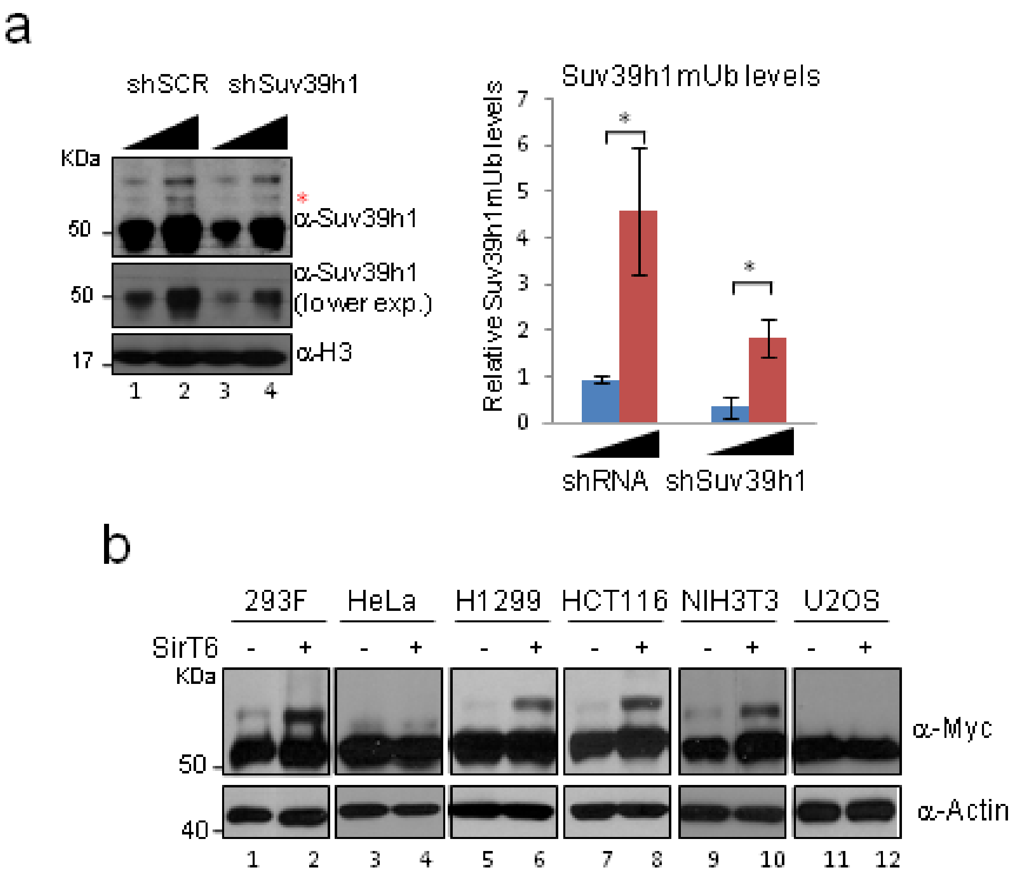


**Supplementary Fig.2. The induction of Suv39h1 modification by SIRT6 is cell-type specific. (a)** Downregulation of Suv39h1 in 293F cells induces a concomitant decrease in the detected endogenous band indicated with a red asterisk. Two different amounts (1x and 2x) of nuclear extract (soluble and benzonase-digested chromatin) of each sample are shown. Samples were previously normalized by histone H3 levels. Left, representative experiment of n=3. Right, quantification and statistical analysis of n=3 experiments. Levels of Suv39h1mUb for each condition are represented relative to the levels of Suv39h1mUb in the first lane of the experiment (shSCR, 1x of nuclear extract described above). (*:p<0.05). **(b)** Western-blot of extracts from the indicated cell lines transfected with myc-Suv39h1 -/+ SirT6-HA as in Fig. 2a.

a

K.AGQPIYEC$_{222}$glyglyNSR.C

| # | b | Seq. | y | # |
|---|---|---|---|---|
| 1 | 72.0444 | A | | 11 |
| 2 | 129.0659 | G | 1280.5688 | 10 |
| 3 | 257.1244 | Q | 1223.5473 | 9 |
| 4 | 354.1772 | P | 1095.4888 | 8 |
| 5 | 467.2613 | I | 998.4360 | 7 |
| 6 | 630.3246 | Y | 885.3519 | 6 |
| 7 | 759.3672 | E | 722.2886 | 5 |
| 8 | 976.4193 | C | 593.2460 | 4 |
| 9 | 1090.4622 | N | 376.1939 | 3 |
| 10 | 1177.4942 | S | 262.1510 | 2 |
| 11 | | R | 175.1190 | 1 |

b

R.C$_{226}$glyglyCCGYDC$_{228}$glyglyPNR.V

| # | b | Seq. | y | # |
|---|---|---|---|---|
| 1 | 218.0594 | C | | 10 |
| 2 | 378.0900 | C | 1258.4398 | 9 |
| 3 | 538.1207 | C | 1098.4091 | 8 |
| 4 | 595.1422 | G | 938.3785 | 7 |
| 5 | 758.2055 | Y | 881.3570 | 6 |
| 6 | 873.2324 | D | 718.2937 | 5 |
| 7 | 1090.2845 | C | 603.2668 | 4 |
| 8 | 1187.3373 | P | 386.2146 | 3 |
| 9 | 1301.3802 | N | 289.1619 | 2 |
| 10 | | R | 175.1190 | 1 |

c

K.AGQPIYECNSR.C

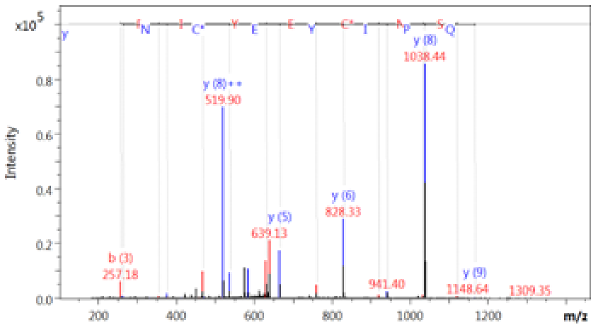


d

K.AGQPIYECNSR.C

| # | b | Seq. | y | # |
|---|---|---|---|---|
| 1 | 72.0444 | A | | 11 |
| 2 | 129.0659 | G | 1223.5473 | 10 |
| 3 | 257.1244 | Q | 1166.5259 | 9 |
| 4 | 354.1772 | P | 1038.4673 | 8 |
| 5 | 467.2613 | I | 941.4145 | 7 |
| 6 | 630.3246 | Y | 828.3305 | 6 |
| 7 | 759.3672 | E | 665.2671 | 5 |
| 8 | 919.3978 | C | 536.2246 | 4 |
| 9 | 1033.4408 | N | 376.1939 | 3 |
| 10 | 1120.4728 | S | 262.1510 | 2 |
| 11 | | R | 175.1190 | 1 |

e

R.CCCGYDCPNR.V

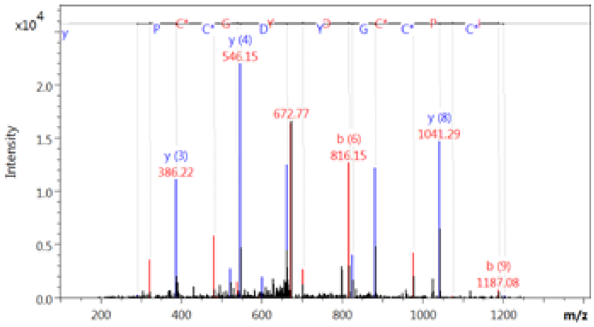


f

R.CCCGYDCPNR.V

| # | b | Seq. | y | # |
|---|---|---|---|---|
| 1 | 161.0379 | C | | 10 |
| 2 | 321.0686 | C | 1201.4183 | 9 |
| 3 | 481.0992 | C | 1041.3877 | 8 |
| 4 | 538.1207 | G | 881.3570 | 7 |
| 5 | 701.1840 | Y | 824.3356 | 6 |
| 6 | 816.2110 | D | 661.2722 | 5 |
| 7 | 976.2416 | C | 546.2453 | 4 |
| 8 | 1073.2944 | P | 386.2146 | 3 |
| 9 | 1187.3373 | N | 289.1619 | 2 |
| 10 | | R | 175.1190 | 1 |

**Supplementary Fig.3. SirT6 promotes cysteine ubiquitination in the PRE-SET domain of Suv39h1. (a)** Table including peptide identification data associated to the MSMS spectra of **K.AGQPIYECN Supplementary Fig.3. SirT6 promotes cysteine ubiquitination in the PRE-SET domain of Suv39h1. SR.C** ubiquitinated in Cys222 shown in Figure 3b (left spectra). Table show y and b fragment series ions observed for each spectra respectively (red numbers indicate observed fragments, black numbers are theoretical fragments). **(b)** Table including peptide identification data associated to the MSMS spectra of **R.CCCGYDCPNR.V** ubiquitinated in Cys226/Cys228 shown in Figure 3b (right spectra). **(c,d)** Non-ubiquitinated **K.AGQPIYECNSR.C** peptide identification data. Peptide MSMS spectra with carbamylated cysteine (precursor m/z 647.7988) (c), and associated table (d). **(e,f)** Non-ubiquitinated **R.CCCGYDCPNR.V** peptide identification data. Peptide MSMS spectra with carbamylated cysteine (precursor m/z 681.2311) (e), and associated table (f).

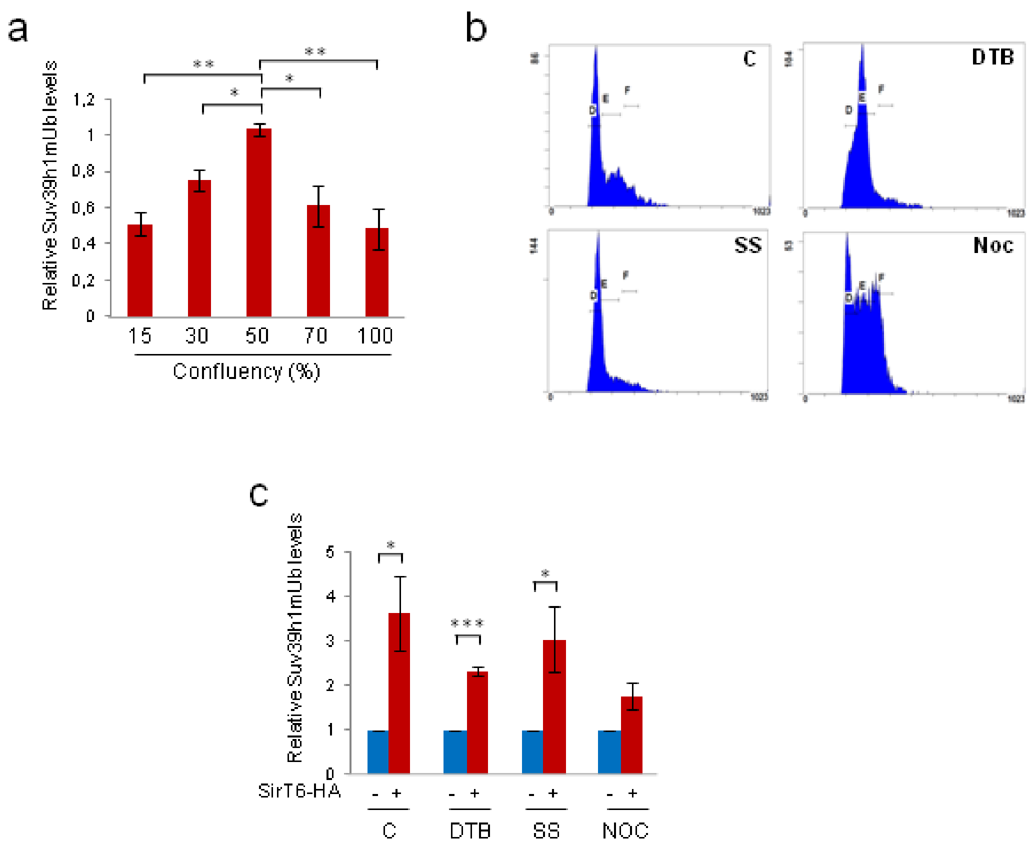


**Supplementary Fig.4**. **Suv39h1 monoubiquitination levels are dependent on proliferation and change during cell cycle (a)** Quantification of n=3 experiments as in Figure 4a. The levels of Suv39h1mUb were normalized with unmodified Suv39h1 levels (lower strong band) and represented relative to 50% confluency, the condition that induced the highest enrichment in the modification. (*: $p<0.05$; **: $p<0.01$). **(b)** FACS analysis of the cell cycle distribution in cells either untreated (C) or treated with double-tymidine block (DTB), Serum starvation (SS) or Nocodazole (NoC) shown in figure 4b. Propidium iodide was use to detect DNA content. In each distribution, D corresponds to $G_1/G_0$ phase, E to S-phase and F to $G_2$/M.mUb in Myc-Suv39h1 expressed, as in Fig. 4b, in the presence or absence of SirT6-HA, but from 293F cells irradiated with 10 Gy. Cells were harvested at the indicated times after irradiation. **(c)** Quantification of n=3 experiments as in Figure 4b. Suv39h1mUb levels were represented similar as in a) but in this case for each condition we represented the Suv39h1mUb levels in presence of SirT6-HA overexpression relative to the levels in absence of SirT6-HA. (*: $p<0.05$; ***: $p<0.005$).

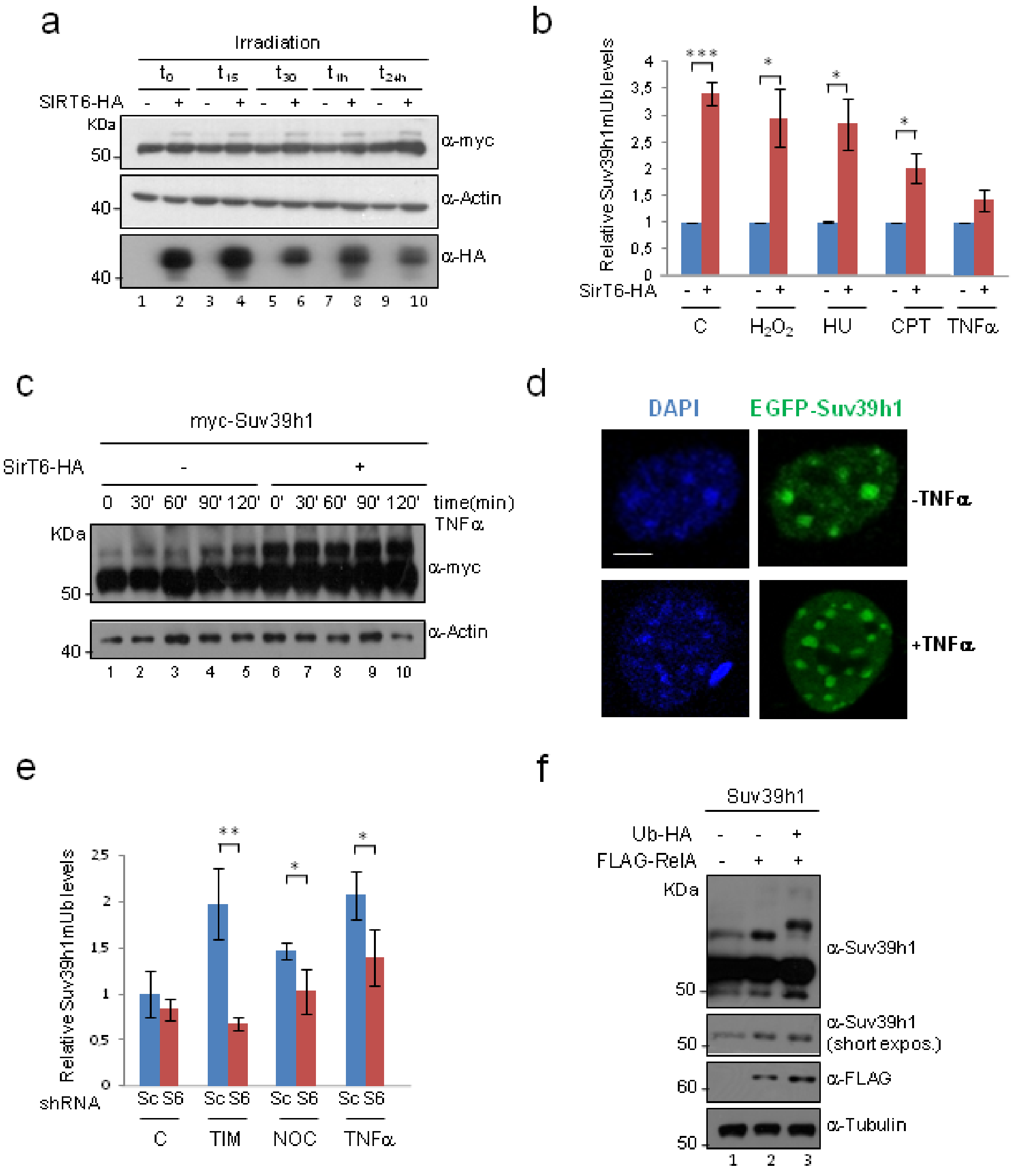


**Supplementary Fig.5. TNFα and RelA induce Suv39h1 monoubiquitination. TNFα activation do not seem to alter Suv39h1 function in Constitutive Heterochromatin (CH) (a)** mUb in Myc-Suv39h1 expressed, as in Fig. 4b, in the presence or absence of SirT6-HA, but from 293F cells irradiated with 10 Gy. Cells were harvested at the indicated times after irradiation. **(b)** Quantification of n=3 experiments as in Figure 4c. Suv39h1mUb levels were represented as in Suppl.Fig.4c. (*: $p<0.05$; ***: $p<0.005$). **(c)** Levels of Suv39h1 mUb in 293F cells under TNFα (20ng/ml) treatment in the indicated times (in minutes). **(d)** Immunofluorescence of Suv39h1-EGFP localization in the CH foci of NIH3T3 cells either untreated or treated with 20ng/ml of TNFα during 1h. DAPI staining is used as a control of DNA and to visualize the CH foci. A representative 5μm scale bar has been included in DAPI –TNFα. **(e)** Quantification of n=3 experiments as in Figure 4e. Suv39h1mUb levels were represented as in b) relative to empty vector transfection (lanes 1,4,7 and 10 of fig 4e) (*: $p<0.05$; **: $p<0.01$). Sc: Shscramble; S6: shSIRT6. **(f)** Experiment as in Figure 2c but overexpressing FLAG-RelA instead of SirT6-HA.

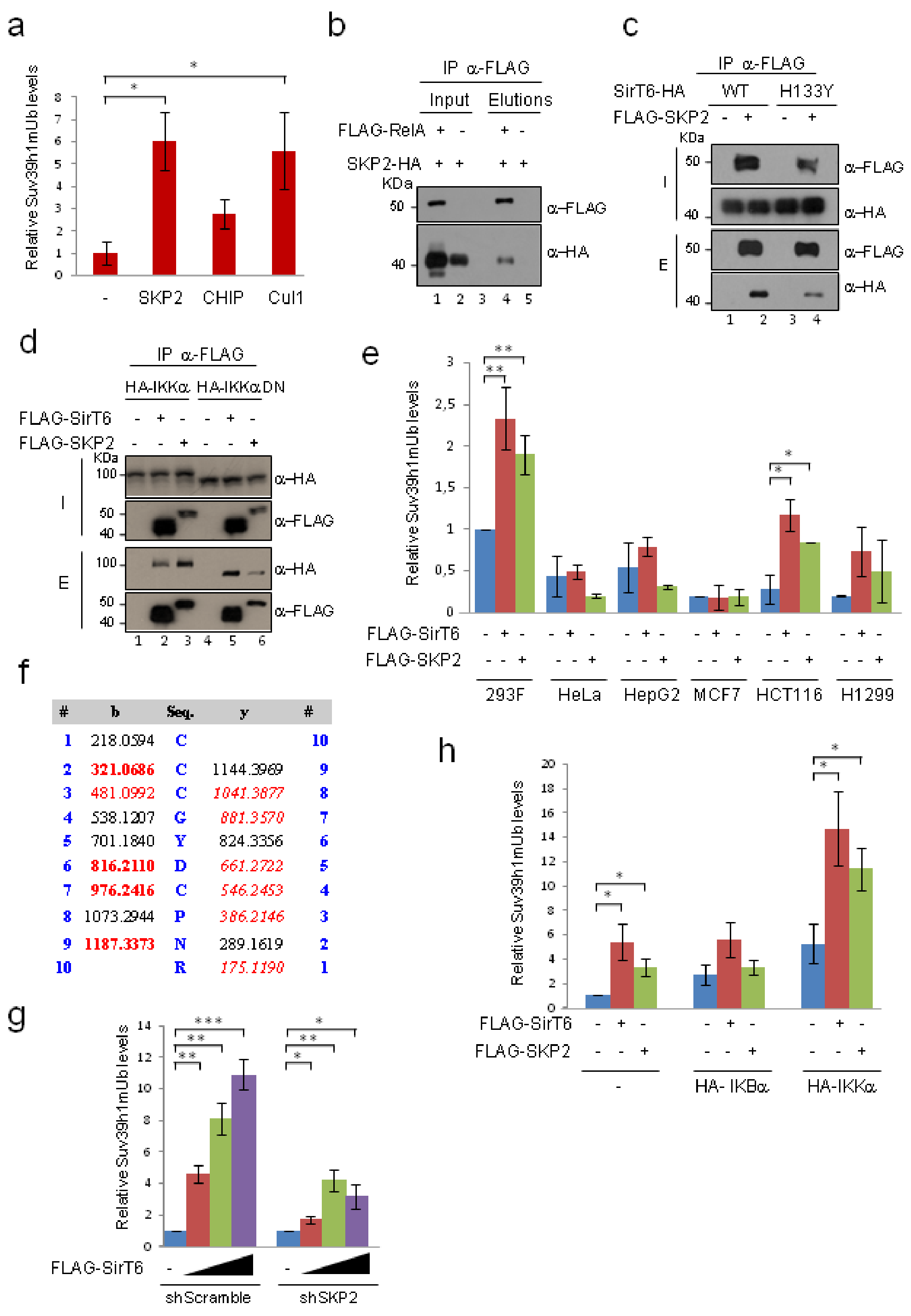


| # | b | Seq. | y | # |
|---|---|---|---|---|
| 1 | 218.0594 | C | | 10 |
| 2 | 321.0686 | C | 1144.3969 | 9 |
| 3 | 481.0992 | C | 1041.3877 | 8 |
| 4 | 538.1207 | G | 881.3570 | 7 |
| 5 | 701.1840 | Y | 824.3356 | 6 |
| 6 | 816.2110 | D | 661.2722 | 5 |
| 7 | 976.2416 | C | 546.2453 | 4 |
| 8 | 1073.2944 | P | 386.2146 | 3 |
| 9 | 1187.3373 | N | 289.1619 | 2 |
| 10 | | R | 175.1190 | 1 |

**Supplementary Fig.6**. **Skp2 induction of mUb in Suv39h1. (a)** Quantification of n=3 experiments as in Figure 5a of Suv39h1mUb normalized with unmodified Suv39h1 and represented relative to mUb levels in empty vector expression (lane 1). (*: p<0.05). **(b)** Immunoprecipitation of FLAG-RelA and Skp2-HA from nuclear extracts of 293F cells using α-FLAG resin as indicated. **(c)** Skp2 binds to SirT6 catalytic-inactive point mutant H133Y. Immunoprecipitation of FLAG-Skp2 with SirT6-HA either WT or mutant H133Y from nuclear extracts of 293F cells using α-FLAG resin. **(d)** Immunoprecipitation from nuclear extracts of 293F of FLAG-SirT6 or FLAG-SKP2 with HA-tagged WT or a catalytic point mutant of IKKα (IKKαDN) using α-HA resin. **e)** Quantification of n=3 experiments as in Figure 5c represented similar suppl.fig 5b but representing all the values of all conditions relative to the levels on mUb in 293F upon expression of empty vector (lane 1). (*: p<0.05). **(f)** Table including peptide identification data associated to the MSMS spectra of **R.CCCGYDCPNR.V** ubiquitinated in Cys226 shown in Figure 5e. **(g)** Quantification of n=3 experiments as in Figure 5g represented similar as e). (*: p<0.05; **: p<0.01; ***: p<0.005). **(h)** Quantification of n=3 experiments as in Figure 5i represented similar as e). (*: p<0.05).

a

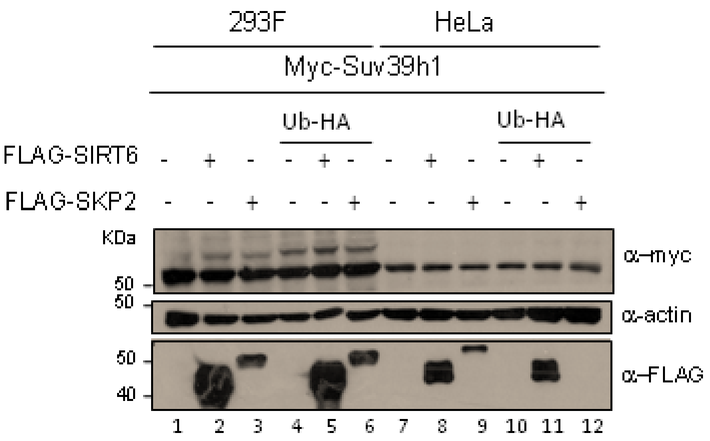


b

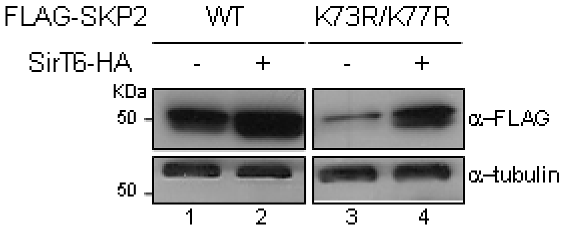


**Supplementary Fig.7**. **Skp2-induced mUb in Suv39h1. (a)** Skp2 induces an ubiquitination in Suv39h1 in 293F cells but not in HeLa cells. Similar experiment as in Figures 2b and Supp. 4e with FLAG-Skp2 and FLAG-SirT6 performed in 293F cells and in HeLa cells. **(b)** The protein levels of Skp2 double mutant K73R/K77R are also upregulated by SirT6. Levels in 293F nuclear extracts of Skp2 either WT or double mutant K73R/K77R in presence or absence of SirT6-HA overexpression as indicated.

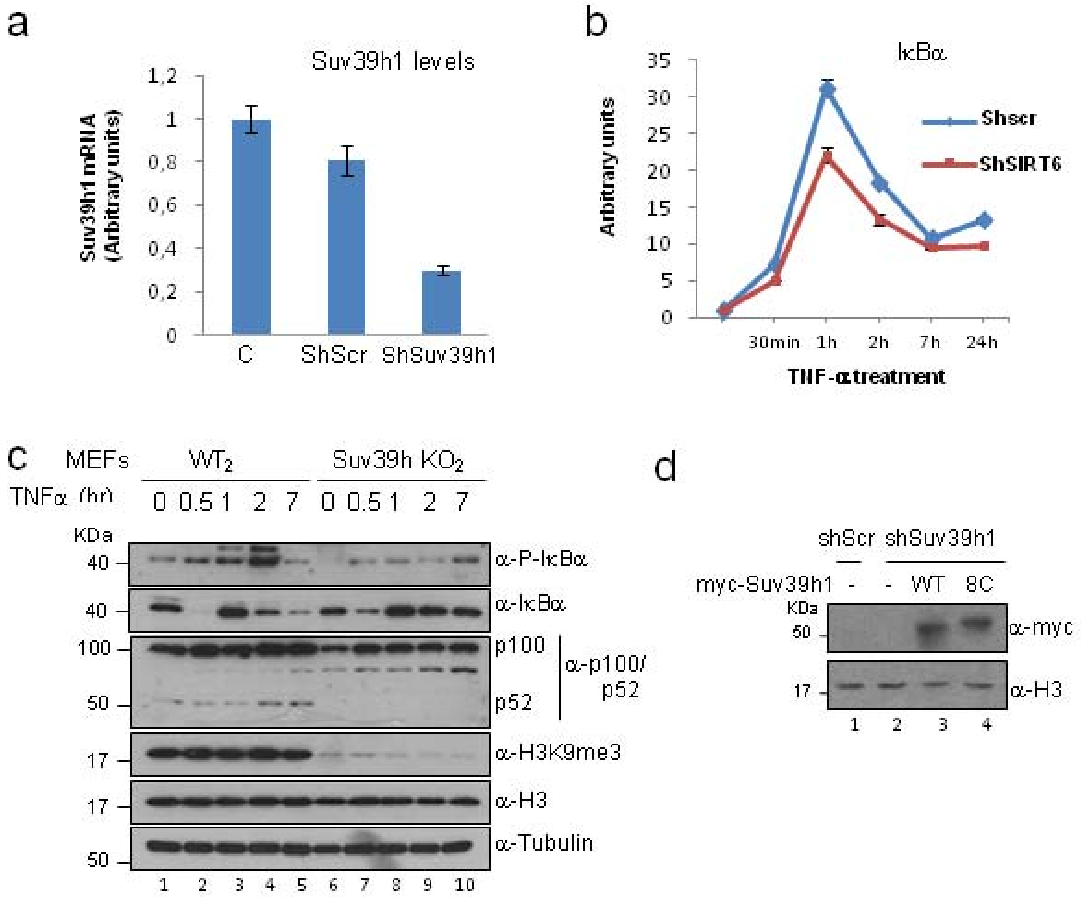


**Supplementary Fig.8**. **Suv39h1 downregulation increases IκBα at RNA and protein levels upon Nf-κB pathway activation by TNFα. (a)** Analysis of the Suv39h1 shRNA used in Figure 7A. Quantitative RT-PCR of the levels of Suv39h1 mRNA in 293F cells upon infection of retrovirus expressing scramble (ShScr) or Suv39h1 ShRNA (ShSuv39h1). **(b)** Profile, as in Figure 7a, of IκBα mRNA levels in 293F cells upon different times of TNFα activation, but infected with a retrovirus expressing scramble (Shscr) or SirT6 ShRNA (ShSIRT6). **(c)** Experiment as in Figure 7c, but using an additional set of WT and KO MEFs ($WT_2$ and Suv39hK$O_2$). **(d)** Levels of expression of WT and 8C Suv39h1 in cells used in Figure 7g.

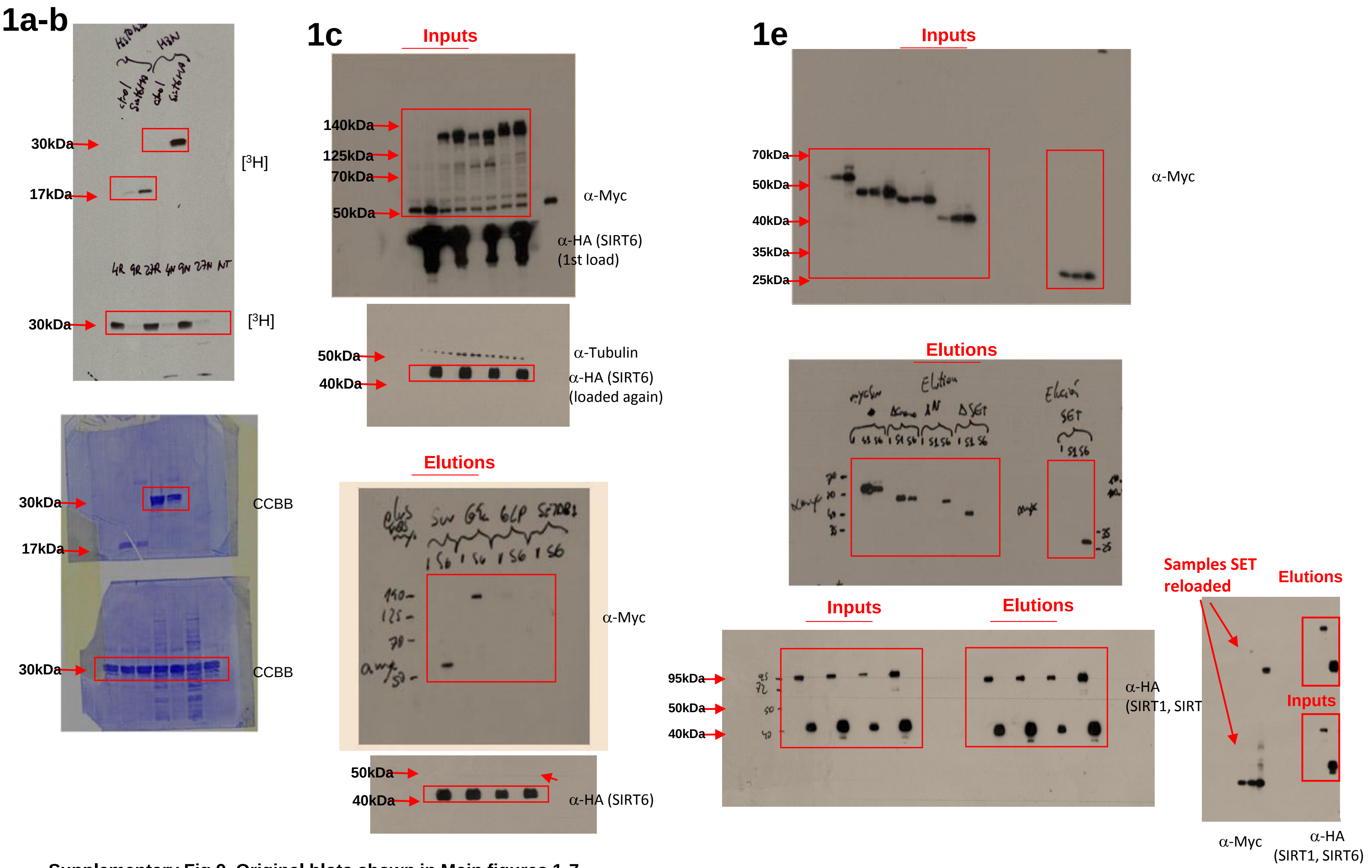


**Supplementary Fig.9. Original blots shown in Main figures 1-7.**

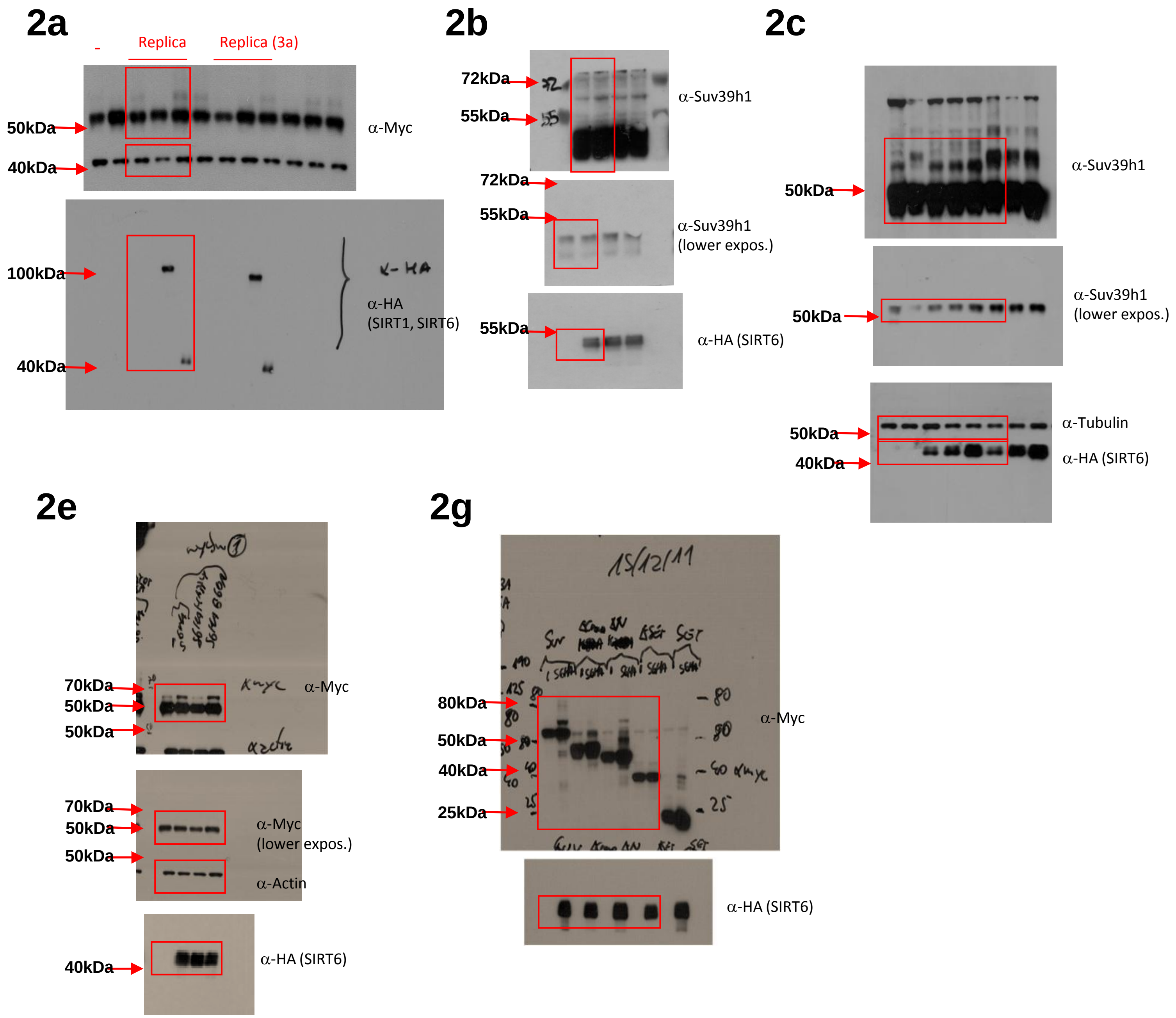
2a
-
Replica
Replica (3a)
50kDa
40kDa
α-Myc
100kDa
40kDa
α-HA
(SIRT1, SIRT6)
2b
72kDa
55kDa
α-Suv39h1
72kDa
55kDa
α-Suv39h1
(lower expos.)
55kDa
α-HA (SIRT6)
2c
50kDa
α-Suv39h1
50kDa
α-Suv39h1
(lower expos.)
50kDa
40kDa
α-Tubulin
α-HA (SIRT6)
2e
70kDa
50kDa
50kDa
α-Myc
70kDa
50kDa
50kDa
α-Myc
(lower expos.)
α-Actin
40kDa
α-HA (SIRT6)
2g
15/12/11
80kDa
50kDa
40kDa
25kDa
α-Myc
α-HA (SIRT6)

## 2h

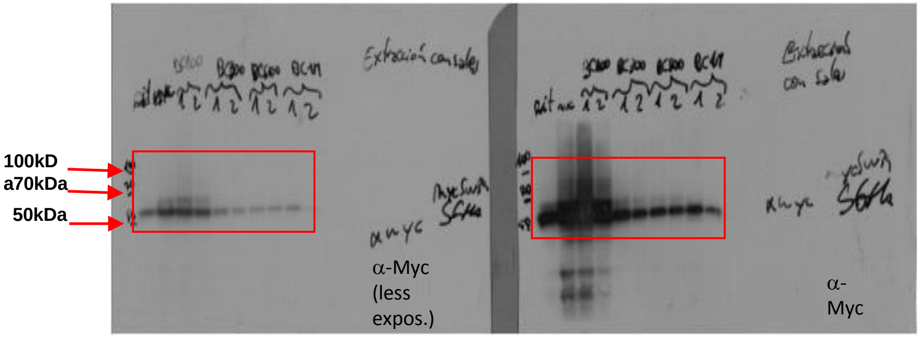
100kD
a70kDa
50kDa
α-Myc
(less
expos.)
α-
Myc

## 2j

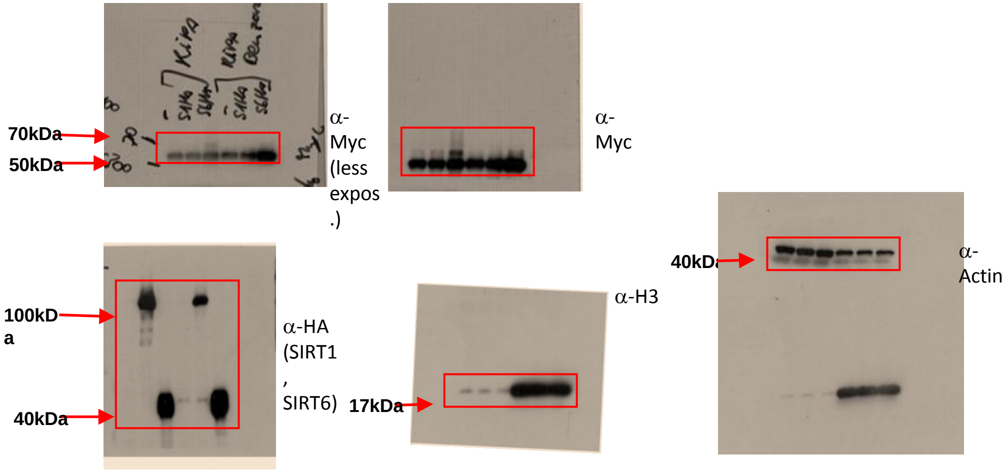
70kDa
50kDa
α-
Myc
(less
expos
.)
α-
Myc
40kDa
α-
Actin
100kD
a
40kDa
α-HA
(SIRT1
,
SIRT6)
17kDa
α-H3

3a

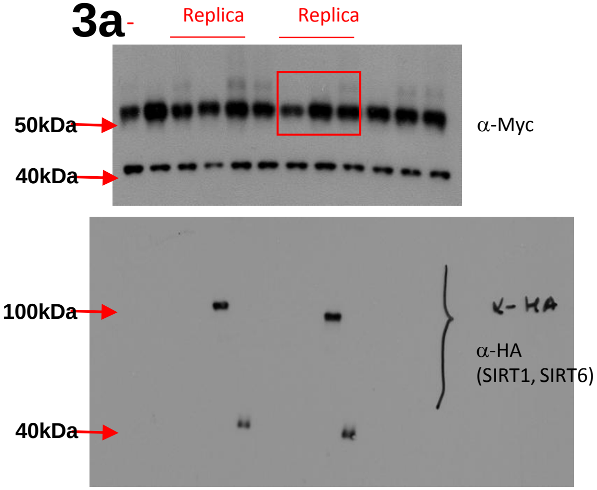

Replica
Replica
50kDa
40kDa
α-Myc
100kDa
40kDa
α-HA
α-HA
(SIRT1, SIRT6)


3d

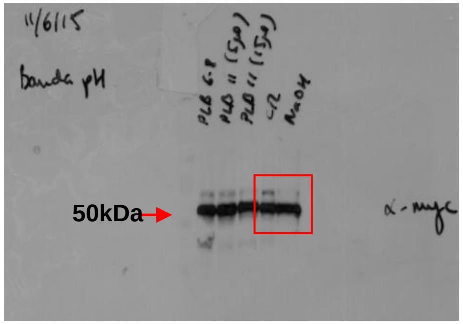

11/6/15
50kDa
α-myc


3f

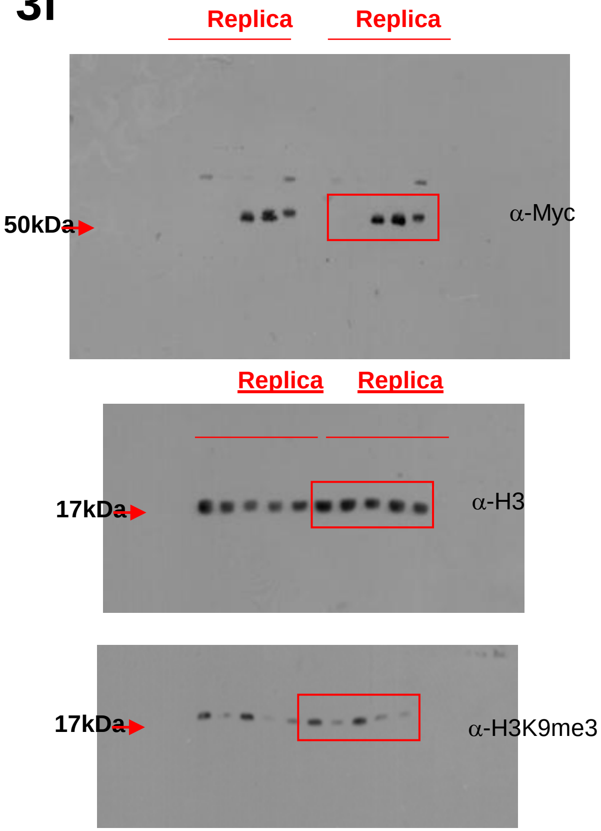

Replica
Replica
50kDa
α-Myc
Replica
Replica
17kDa
α-H3
17kDa
α-H3K9me3

4a

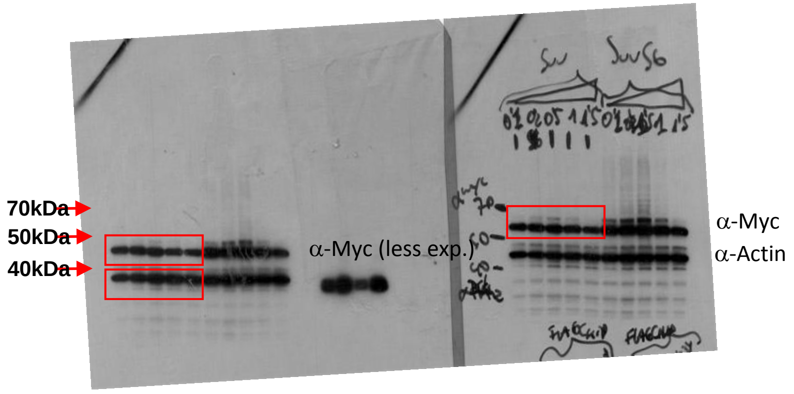
70kDa
50kDa
40kDa
α-Myc (less exp.)
α-Myc
α-Actin

4b

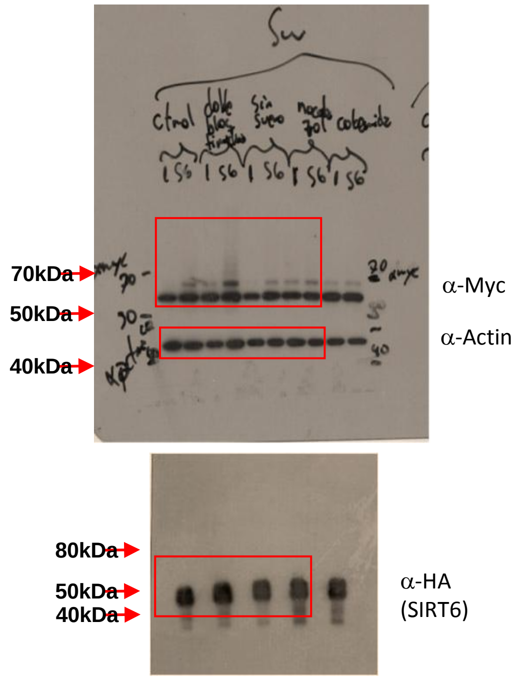
70kDa
50kDa
40kDa
α-Myc
α-Actin
80kDa
50kDa
40kDa
α-HA
(SIRT6)

4c

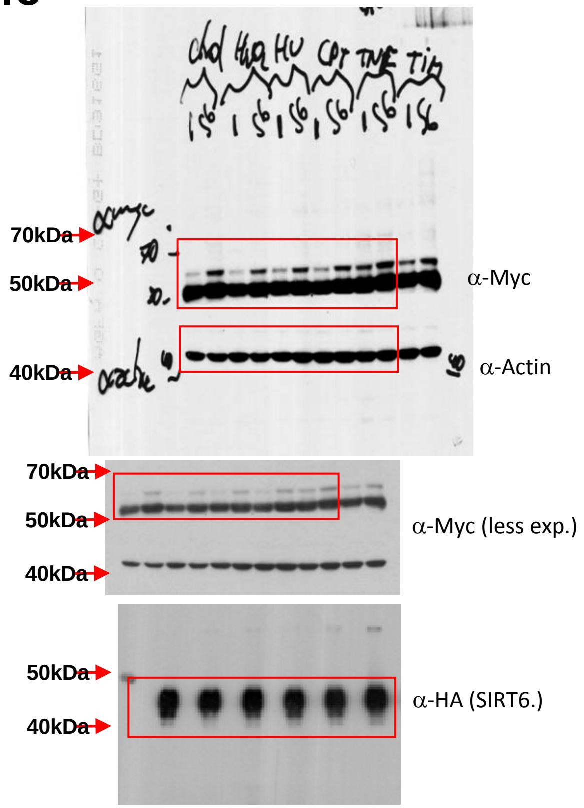
70kDa
50kDa
40kDa
α-Myc
α-Actin
70kDa
50kDa
40kDa
α-Myc (less exp.)
50kDa
40kDa
α-HA (SIRT6.)

4d

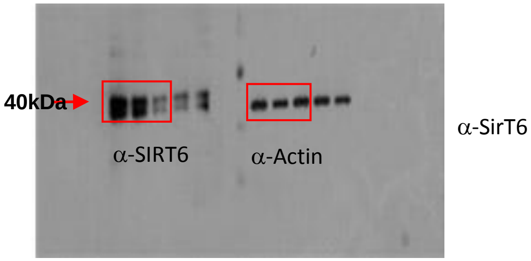
40kDa
α-SIRT6
α-Actin
α-SirT6

4e

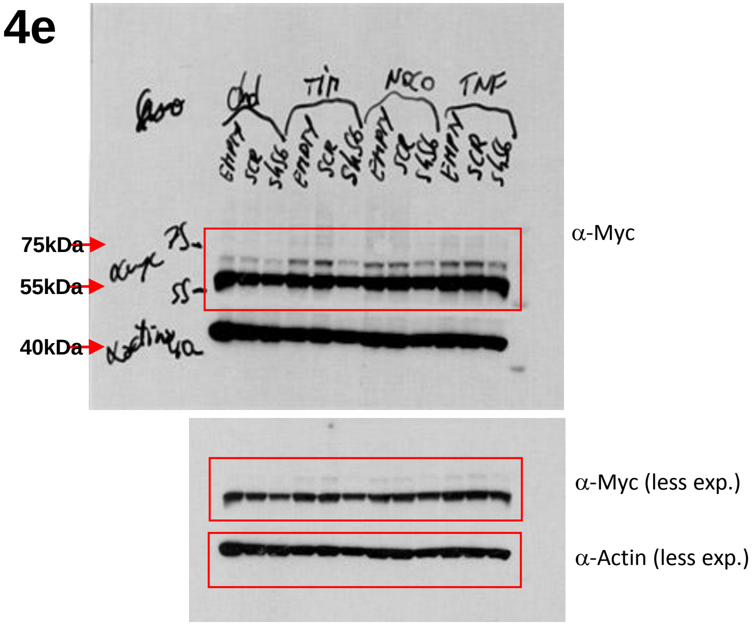
75kDa
55kDa
40kDa
α-Myc
α-Myc (less exp.)
α-Actin (less exp.)

4f

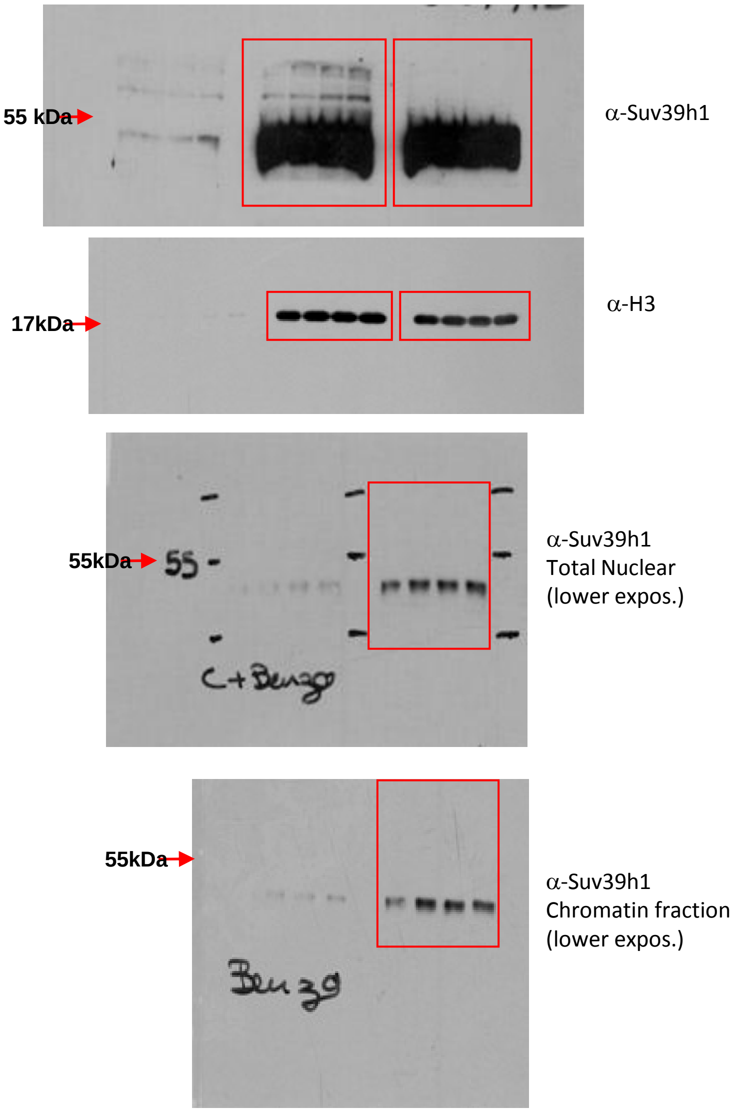
55 kDa
α-Suv39h1
17kDa
α-H3
55kDa
α-Suv39h1
Total Nuclear
(lower expos.)
55kDa
α-Suv39h1
Chromatin fraction
(lower expos.)

4g

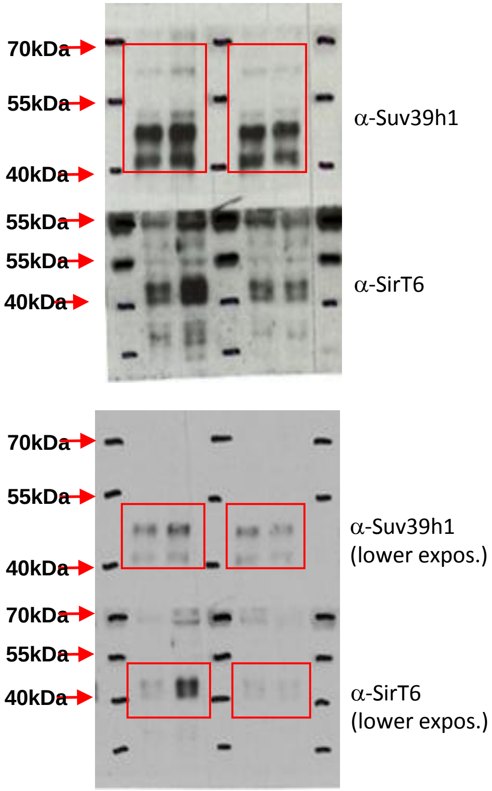
70kDa
55kDa
40kDa
α-Suv39h1
55kDa
55kDa
40kDa
α-SirT6
70kDa
55kDa
40kDa
α-Suv39h1
(lower expos.)
70kDa
55kDa
40kDa
α-SirT6
(lower expos.)

4h

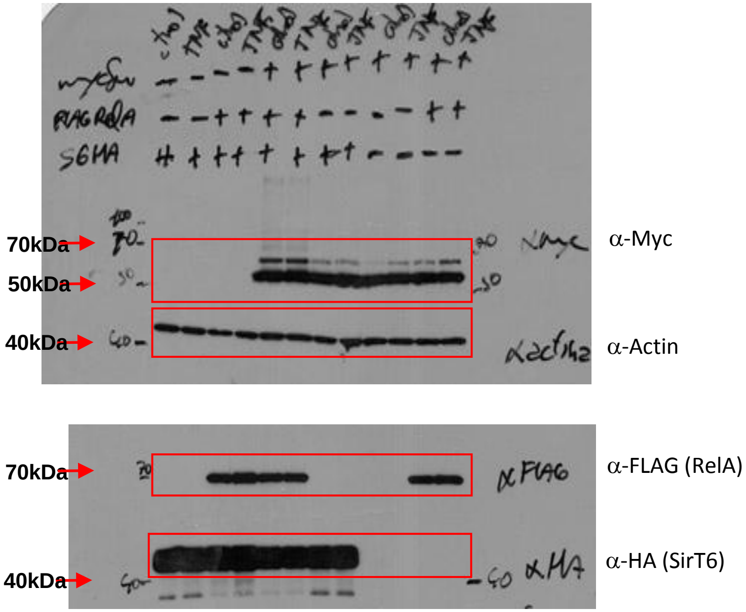
70kDa
50kDa
α-Myc
40kDa
α-Actin
70kDa
α-FLAG (RelA)
40kDa
α-HA (SirT6)

4i

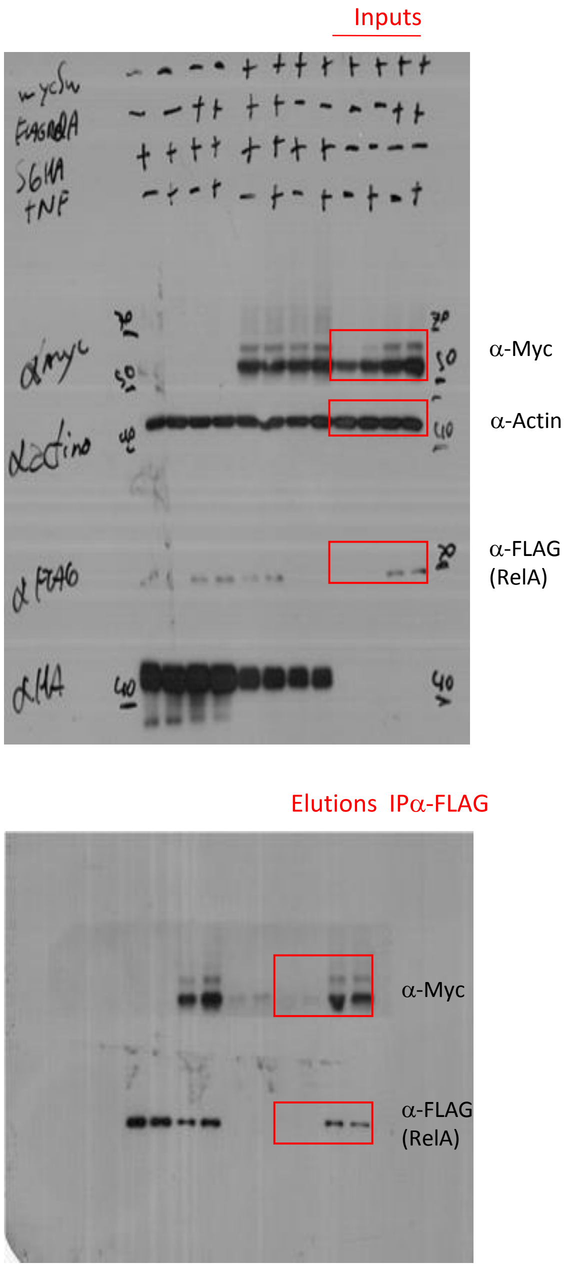
Inputs
α-Myc
α-Actin
α-FLAG
(RelA)
Elutions IPα-FLAG
α-Myc
α-FLAG
(RelA)

5a

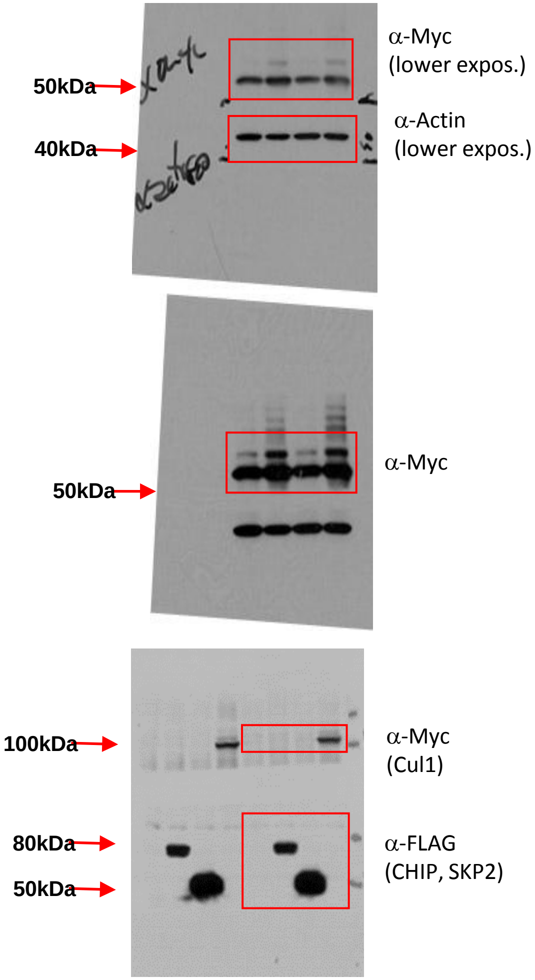
50kDa
40kDa
α-Myc
(lower expos.)
α-Actin
(lower expos.)
50kDa
α-Myc
100kDa
α-Myc
(Cul1)
80kDa
50kDa
α-FLAG
(CHIP, SKP2)

## 5b

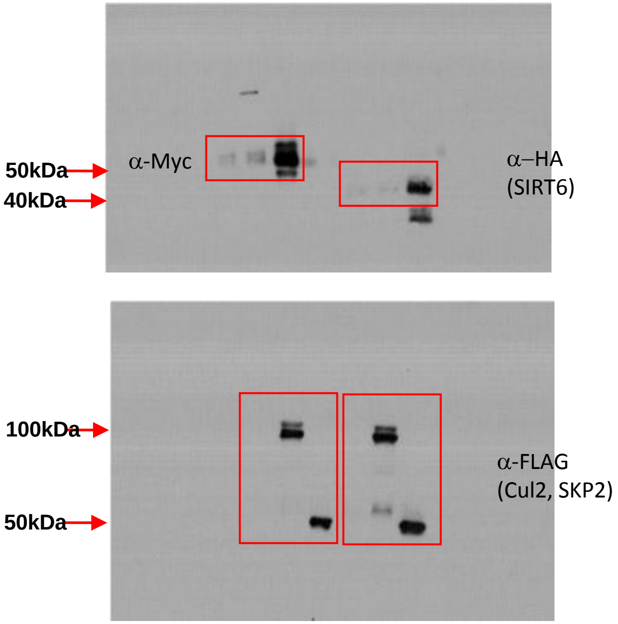

50kDa
40kDa
α-Myc
α–HA
(SIRT6)
100kDa
50kDa
α-FLAG
(Cul2, SKP2)


## 5c

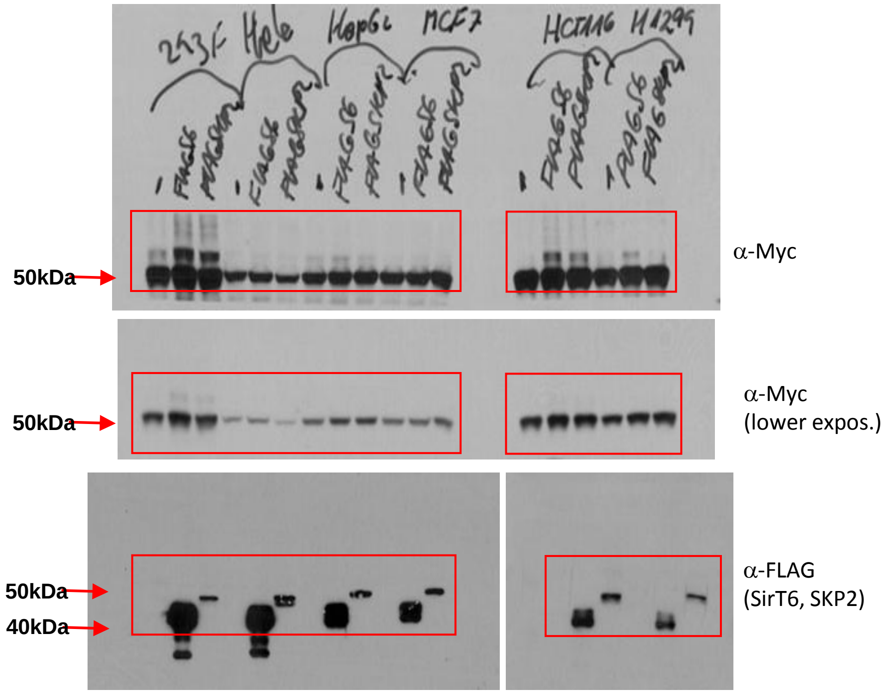

50kDa
α-Myc
50kDa
α-Myc
(lower expos.)
50kDa
40kDa
α-FLAG
(SirT6, SKP2)

**5d**

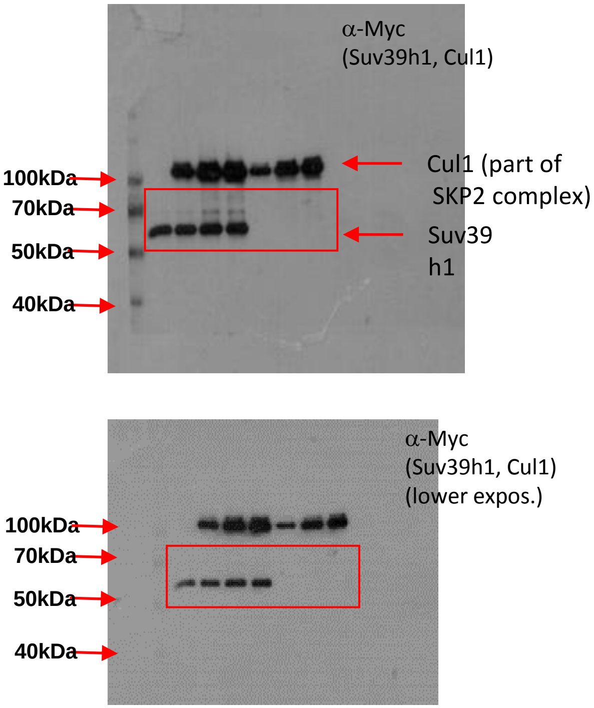
α-Myc
(Suv39h1, Cul1)
100kDa
70kDa
50kDa
40kDa
Cul1 (part of
SKP2 complex)
Suv39
h1
α-Myc
(Suv39h1, Cul1)
(lower expos.)
100kDa
70kDa
50kDa
40kDa

**5f**

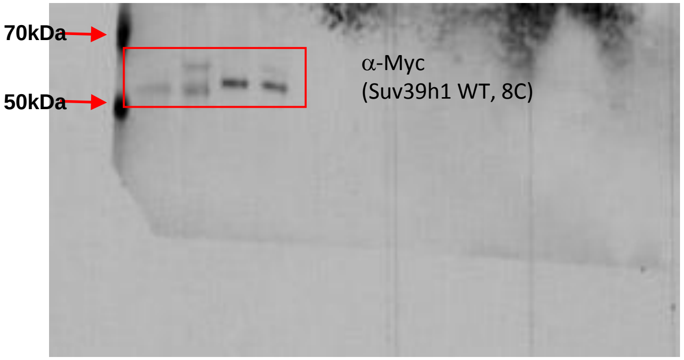
70kDa
50kDa
α-Myc
(Suv39h1 WT, 8C)

**5g**

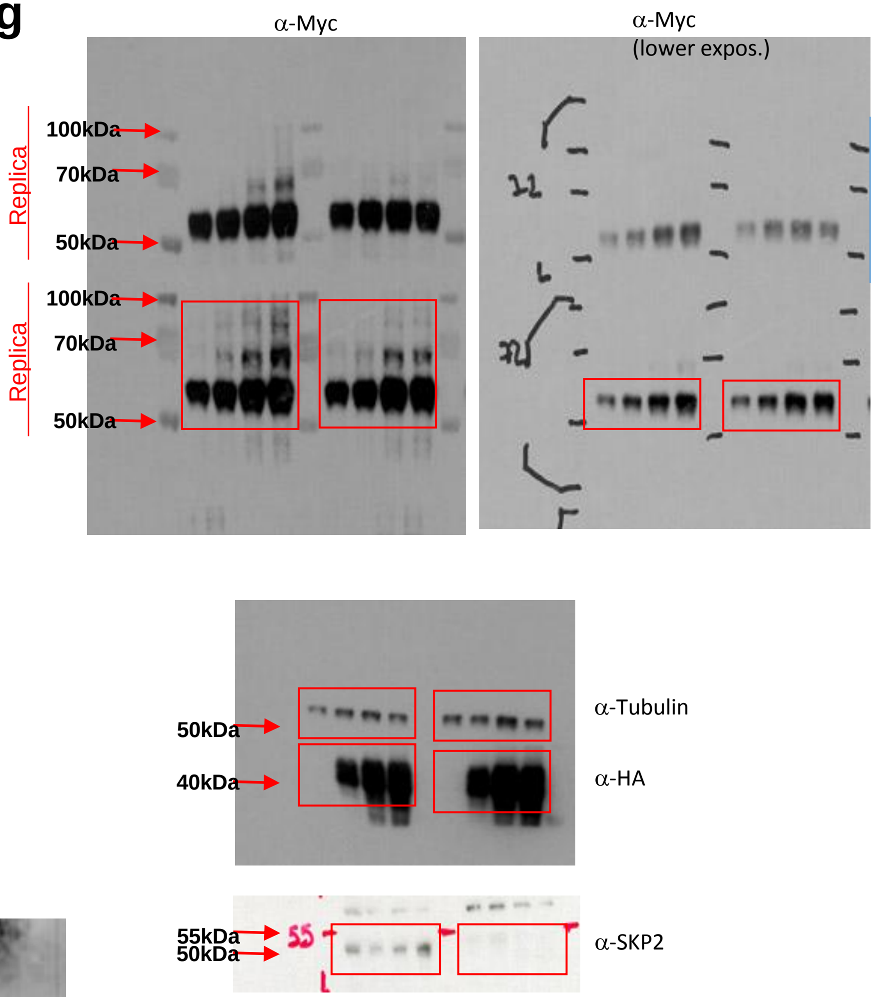
α-Myc
α-Myc
(lower expos.)
Replica
100kDa
70kDa
50kDa
Replica
100kDa
70kDa
50kDa
50kDa
40kDa
α-Tubulin
α-HA
55kDa
50kDa
α-SKP2

5h

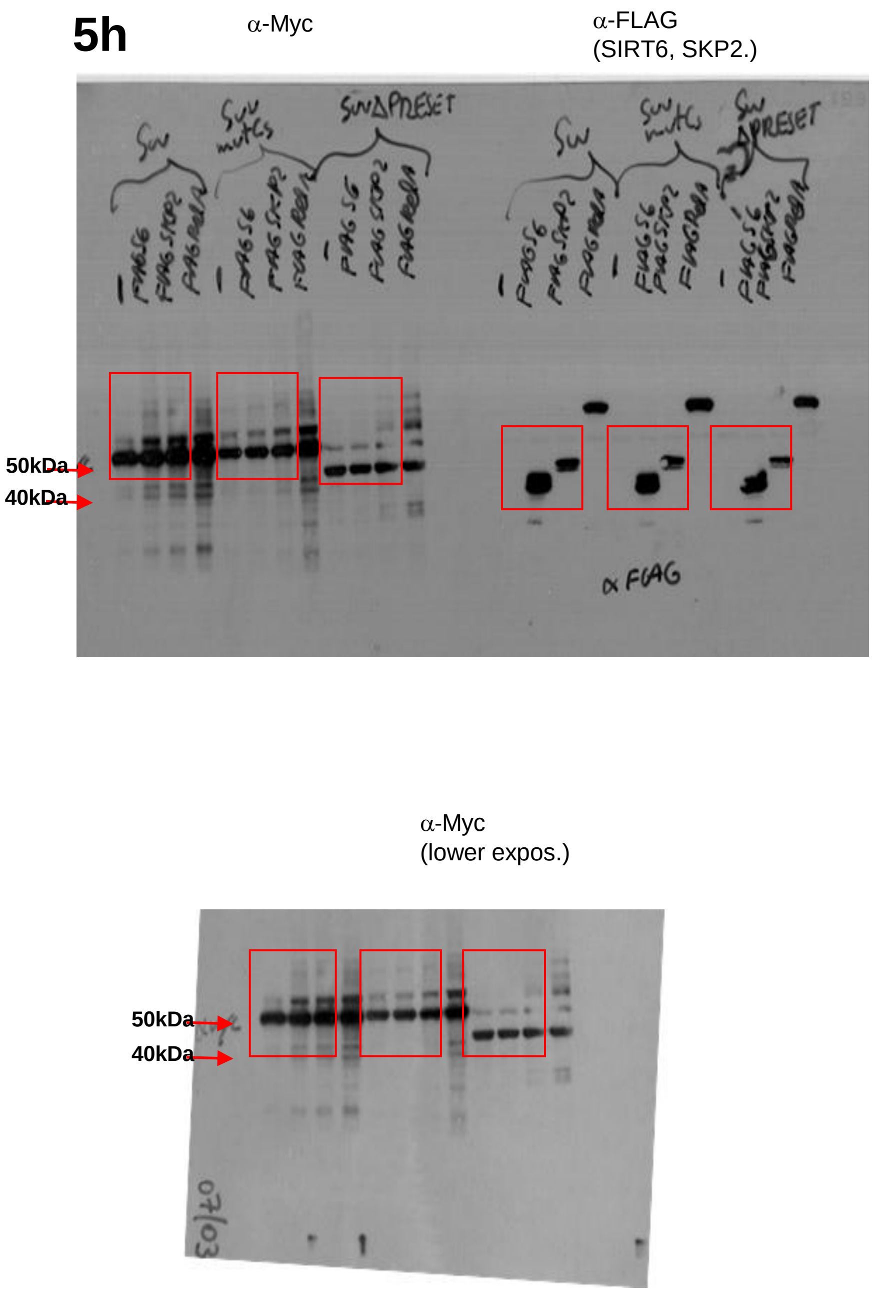
α-Myc
α-FLAG
(SIRT6, SKP2.)
50kDa
40kDa
α FLAG
α-Myc
(lower expos.)
50kDa
40kDa

5i

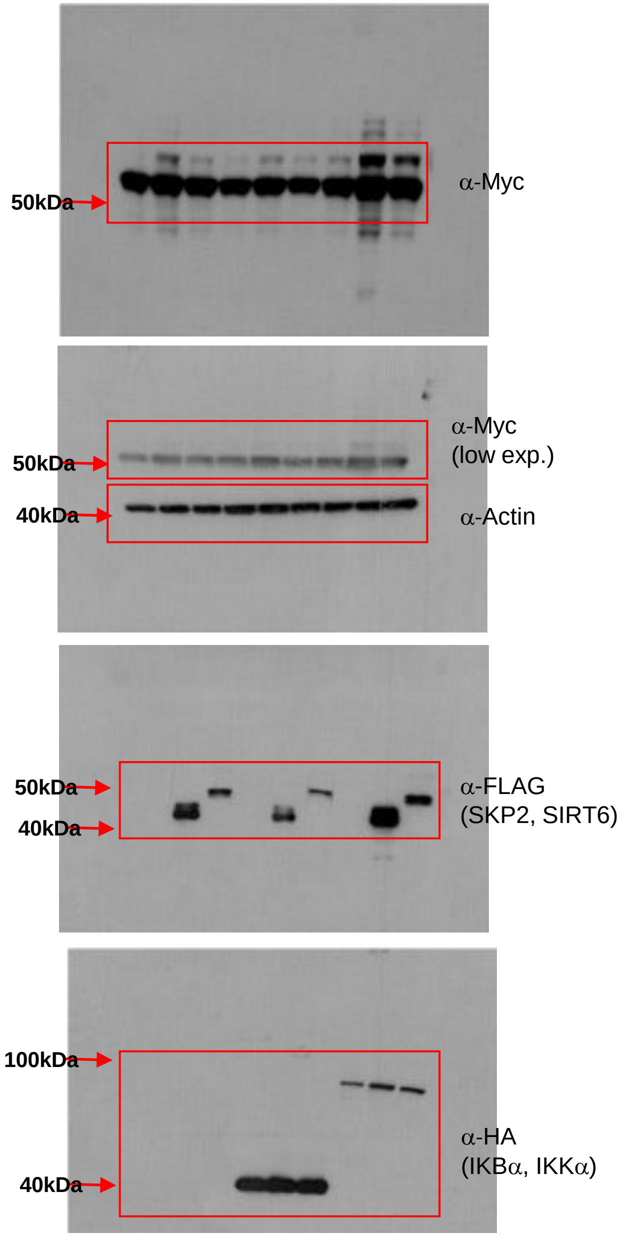
50kDa
α-Myc
α-Myc
(low exp.)
50kDa
40kDa
α-Actin
50kDa
40kDa
α-FLAG
(SKP2, SIRT6)
100kDa
40kDa
α-HA
(IKBα, IKKα)

## 6a (293F)

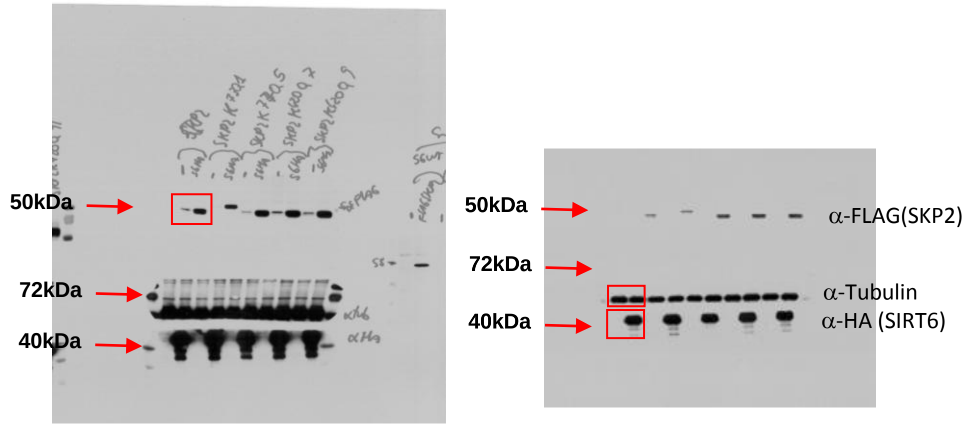


## 6a(HeLa)

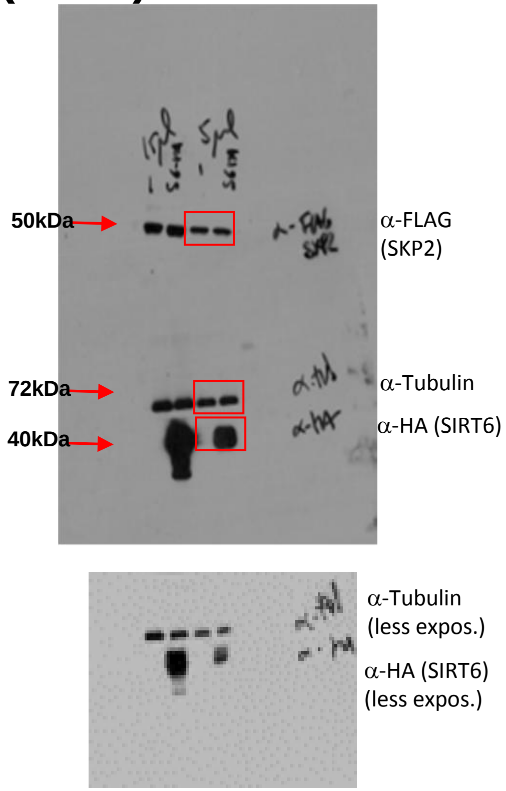


## 6b (shRNA)

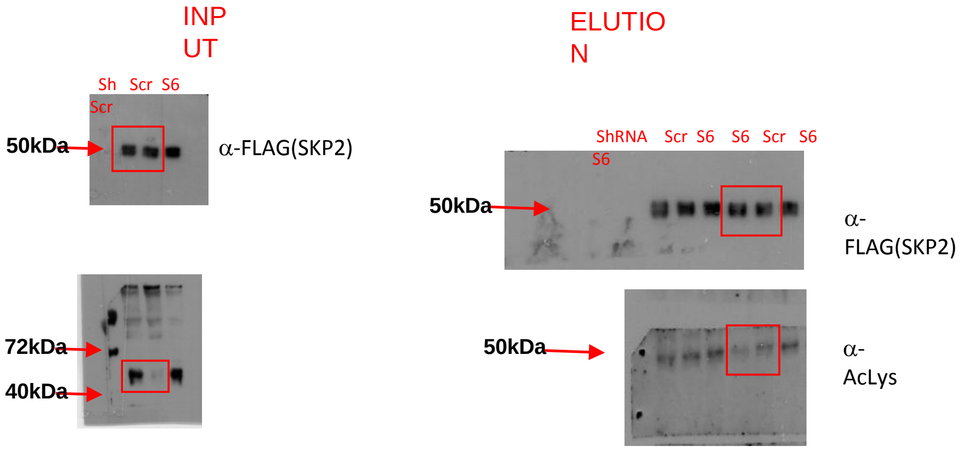

**6b (overex.)**

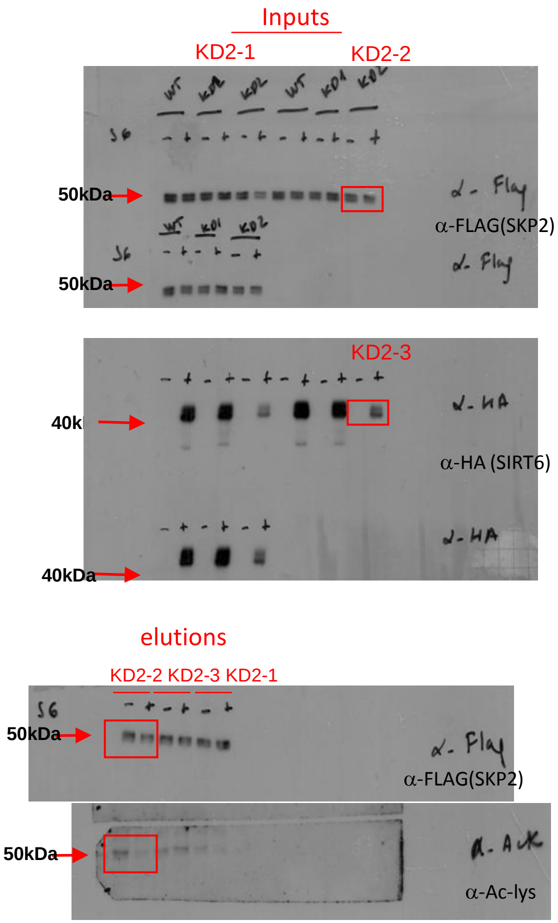


**6e**

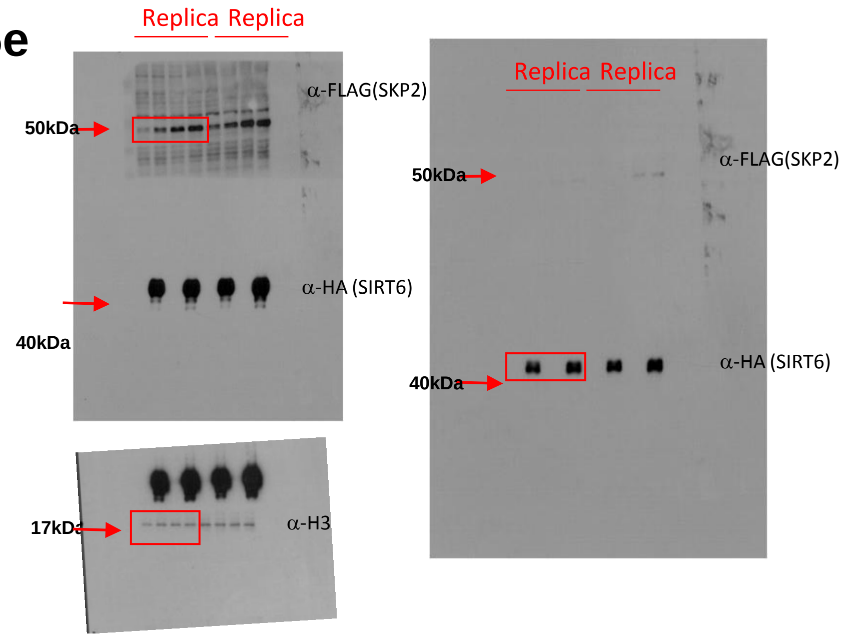


**6g**

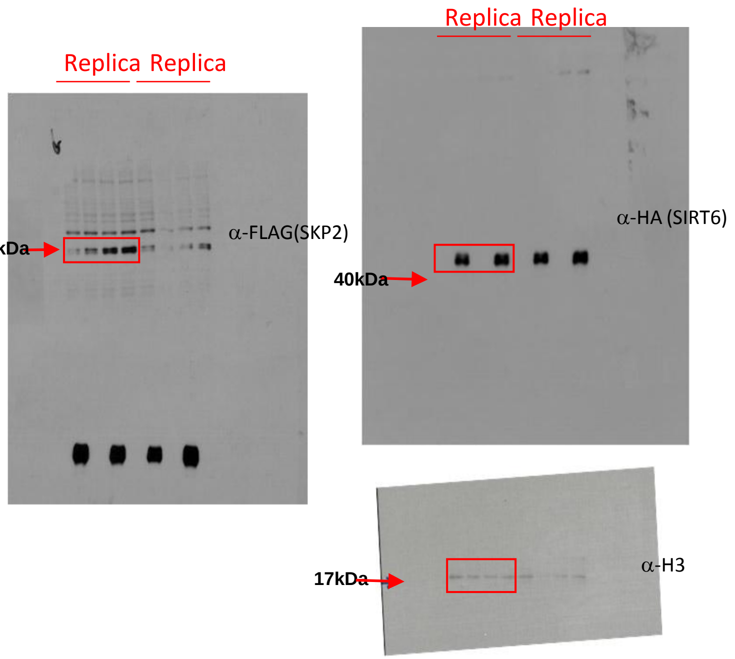

7c

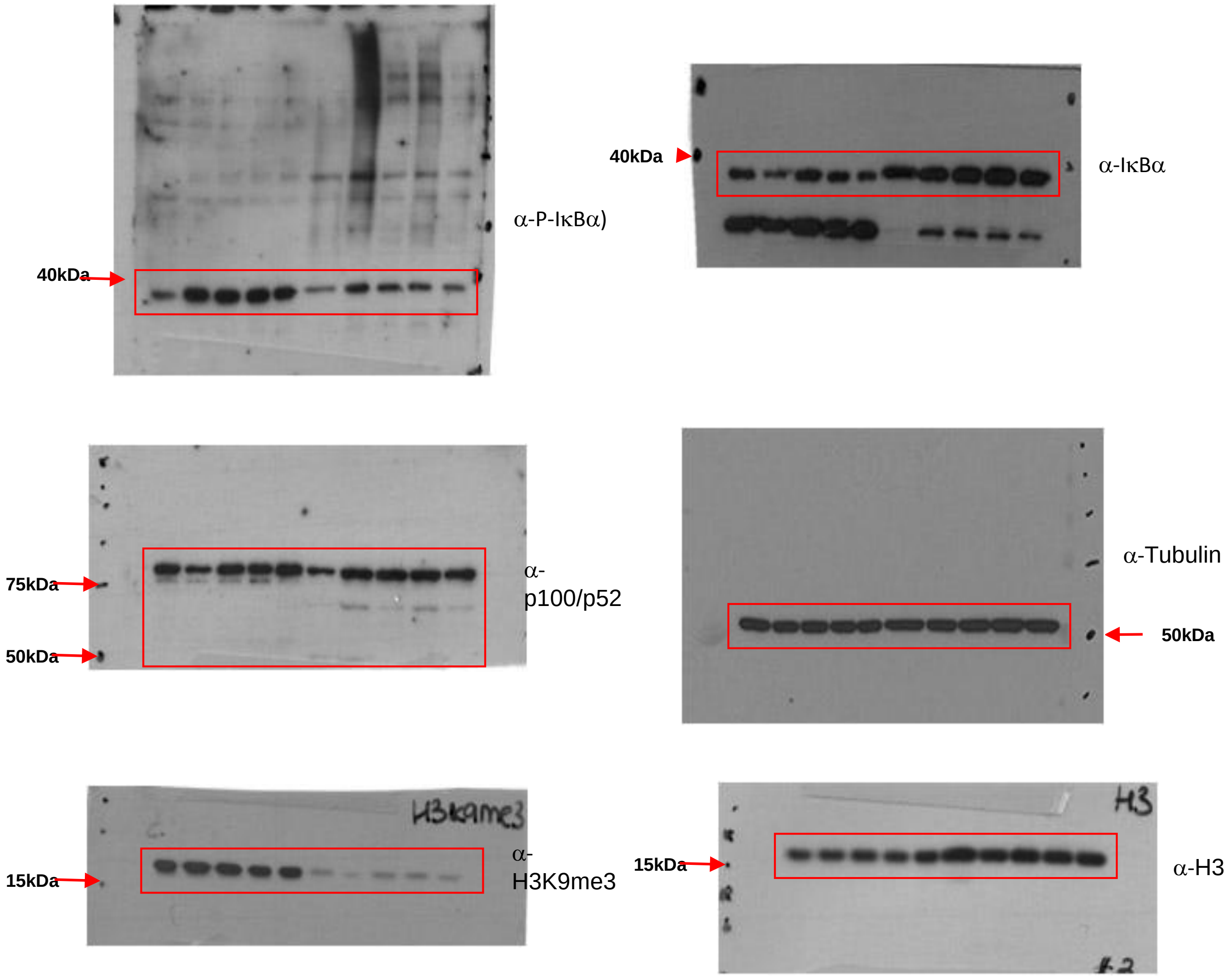

40kDa
α-P-IκBα)
40kDa
α-IκBα
75kDa
50kDa
α-p100/p52
α-Tubulin
50kDa
H3k9me3
15kDa
α-H3K9me3
H3
15kDa
α-H3
#2

## Supplementary Information

## Supplementary Methods

### Plasmids

SirT6-HA was subcloned from pcDEFSirT6-FLAG (a gift from Dr E. Verdin, Gladstone Inst, San Francisco, USA) into pcDNAT0 HA, by using the primers EcoRISirt6U and SirT6HA-NotI described in the Table S3. Point mutants SirT6-HA H133Y and G60A were generated by directed mutagenesis using the indicated primers. SirT1-HA was subcloned from FLAG-SirT1 vector[1] in pcDNA4T0-HA using the primers SirT1BamHIU and SirT1NotIL described in Table S3.

Myc Suv39h1 and the rest of myc-tagged constructions ΔN, Δchromo and ΔSET were described before[2]. Myc-SET construction was cloned in the same vector using the primers SuvmycSETNotIU and SuvmycSETXhoIL. Myc-SETΔC mutant was generated from the Myc-SET vector by introducing a STOP codon in the last residue of the SET domain (corresponding to residue 370 of Suv39h1) using the primers deltaCmycSuv39U and deltaCmycSuv39L. ΔPRESET construction was generated from the mycSuv39h1 vector by creating through directed mutagenesis (primers BamHIdeltaPRESET and BamHIdeltaPRESETfin) two BamHI sites flanking PRESET sequence, digesting with BamHI and religating the vector. Regarding Suv39h1-expressing retrovirus, mycSuv39h1 sequence was cloned in the puromycin-resistant retroviral vector pMSCV using the primers PMSCVmycSuv39h1F and PMSCVmycSuv39h1R. The 3C mutant (Cys residues C222, C226 and C232 mutated to Ala) of Suv39h1 was generated by directed mutagenesis with PRESETmutCAU and PRESETmutCAL primers. The 8C mutant (Cys residues C181, C183, C186, C194, C195, C222, C226 and C232 mutated to Ala) was generated with directed mutagenesis in sequential steps from the 3C mutant, then to a 5C mutant (with PRESETC818386AU and PRESETC818386AU primers), and then finally to the 8C mutant (with PRESETC9495AU and PRESETC9495AL primers).

MycG9a and mycGLP were subcloned in pcDNA3.1myc from the expression vectors pRev G9a long E411and pRev GLP E412 (a kind gift from Dr Ait-Si-Ali, UMR7216, Paris Diderot, France) using the primers mycG9aEcoRIU and mycG9aXhoIL for G9a, and mycGLPXbaIU and mycGLPXbaIL for GLP. Myc SETDB1 expression vector was a gift from Dr G. Pfeifer, BRI, Duarte, USA). FLAG-SKP2 and FLAG-SKP2 K68R/K71R [3] were kindly provided by Dr W. Wei (Harvard MS, Boston, USA). FLAG-SKP2 K68/71/73/77R and FLAG-SKP2 S72D S75D were generated from these vectors by directed mutagenesis using the indicated primers. Expression vector pcDNA3-myc3-

CUL1[4] and Flag-Cul2[5] were a gift from Yue Xiong (UNC, Chapel Hill, USA) (Addgene plasmid # 19896) and Dr T. Hagen (NYS, Singapore), respectively. HA-Skp1 and Myc-Roc1 expression vectors[6] were kindly provided by Dr Marcelo D. Gomes (U.São Paulo, Brazil). FLAG-CHIP was cloned in PCMV4-FLAG (Sigma-Aldrich) from a myc-CHIP expression vector kindly provided by Dr C. Patterson (UNC, Chapel Hill, USA) using the primers CHIPFLAGEcoRIU and CHIPFLAGBamHIL[7]. The expression vectors for IKKα and IKKαDN were described before [8]. IkBα-HA expression vector was generated by subcloning IkBα cDNA from pBabe-Puro-IKBalpha-wt (a gift from William Hahn (Dana Farber Cancer Inst., Boston, USA) , Addgene plasmid # 15290)[9] into the pcDNA4T0-HA vector by the primers IKbaBamHIU and IKbaNotIL.

The shRNA vector pLKO1 shSuv39h1 vector was provided by Dr D'Adda Di Fagagna and was described before[10]. pLKO1 shSirt6 was cloned in pLKO1 vector based on the sequence described in Kawahara et al., 2009 using the primers described in supplementary Table S3. The plKO1 Scrambled was purchased in Addgene (a gift from David Sabatini , Addgene, #1864)[11]. shRNA vectors of SKP2 were previously described [12].

**Protein digestion, LC-MSMS analysis and protein sequence database searching**

Gel bands corresponding to the protein of interest (Suv39h1 or SKP2) were manually excised and trypsin digested. In-gel digestion was carried out following the protocol described by Shevchenko *et al* with minor modifications[13]. Briefly, gel bands were washed with water, ammonium bicarbonate (50 mM) and 50% acetonitrile. Next, samples were reduced by incubation with dithiothreitol (10mM) at 60 °C for 45 min and alkylated with cloracetamide and iodocetamide (50mM) for 30 min in the dark[14]. Finally, proteins were digested with trypsin (13ng/ul) for 3 h at 37 °C (Trypsin gold, Promega). Digestion was stopped by addition of 5% formic acid and peptides extracted twice with 70%acetonitrile 5% formic acid (10min sonication). Peptide extracts were evaporated to dryness, resuspended with 2% acetonitrile 0,1% formic acid and analyzed by nano-HPLC-MSMS.

Peptides were analyzed using an Easy-nanoLCII (Proxeon, Denmark) coupled to an Amazon ETD Ion trap (Bruker Daltonics, Bremen, Germany). Peptides were first trapped on an Easy column TM C18 (2cm, 5µm, 100 µm ID, Thermo Scientific), and then separated using an analytical C18 nanocapillary column (75µM ID, 15 cm, Acclaim®PepMap 100 Thermo Scientific). The chromatograph gradient was achieved by increasing percentage of buffer B from 0–35% at a flow rate of 300 nL/min over 40 or 60 minutes (A: 0.1% formic acid, B: 0.1% formic acid 100% acetonitrile). Eluted peptides were then introduced into Amazon ETD Ion trap by electrospray ionization in

the Captive-Spray ion Source (Bruker Daltonics) with an applied voltage of 1450V and N2 as drying gas. Peptides masses (400-1400 m/z) were analyzed at full scan at enhanced resolution (range 50-3000 m/z and speed 8.100 m/z sec) and 10 most intense peptides were selected and fragmented in a three-dimensional ion trap using both collision induced dissociation (CID) and electron transfer dissociation (ETD) fragmentation, using as a collision gas hellium and methane, respectively. Data was generated with Data Analyst 4.1 software (Bruker Daltonics). In some cases peptides were also analyzed using an EASY-nLC system coupled to a high resolution LTQ Velos-Orbitrap (Thermo Fisher Scientific, Bremen, Germany). Peptides were trapped and separated using an analytical C18 column (75µM ID, 10 cm, Acclaim®PepMap 100 Thermo Scientific) in a gradient from 0-35% at 300 nL/min over 30 minutes. Eluted peptides were subjected to electrospray ionization in an emitter needle (stainless steel nano-bore Proxeon, Thermo Fisher Scientific) with an applied voltage of 1.9kV. Peptide masses (m/z 300-1600) were analyzed in data dependent mode where a full Scan MS was acquired in the Orbitrap with a resolution of 30,000 FWHM; up to the 20 most abundant peptides were selected from each MS scan and then fragmented using CID in the linear ion trap using helium as collision gas.

MS and MS/MS raw data were analyzed with Protein Scape 3.1.2 software (Bruker Daltonics) using Mascot 2.4.0 (Matrix Science) as the search engine and SwissProt database (2013-10, 541,561 sequences; 192,480,382 residues). The specific parameters for protein sequence database searching included taxonomy Mus musculus (20,278 sequences), trypsin digestion with 2 missed cleavages, charge states +1, +2 +3 for precursor ion, mass error of 100 ppm for precursor ion and 0,5 Da for fragment ions (20 ppm and 2Da for Orbitrap data). To detect ubiquitinated peptides we search for the glygly residue in cysteine, lysine, serine and threonine, methionine oxidation and cysteine carbamylation as variable modifications. To detect phosphorylated and acetylated peptides parameters settings were cysteine carbamylation as fixed modification, serine, threonine and tyrosine phosphorylation, lysine acetylation and methionine oxidation as variable modifications. For the identification a significance threshold peptide decoy (Mascot) of $p<0.05$ was set and only peptides with a minimum Mascot score of 25 and proteins with a minimum Mascot score of 35 were considered. Spectra from glygly modified ubiquitinated, phosphorylated and acetylated peptides were also manually examined. The data corresponding to both glygly identification (Suv39h1) and Phosphoserine/acetyl-lysine (SKP2) are included in Tables S1 and S2, respectively.

**Supplementary Table Legends**

**Supplementary Table 1. MS analysis of Suv39h1mUb promoted by SIRT6 and SKP2.** The file includes cysteine gly-gly signature identified upon SIRT6 (1) or SKP2 incubation (2). The peptides included in both tables represent the detected Suv39h1 peptides identified in the Suv39h1 mUb band upon expression of both proteins. In the same experiment, in the absence of SIRT6 or SKP2 we did not detect any Suv39h1 band corresponding to mUb. Accordingly, MS analysis of the area of the gel equivalent to mUb did not render any Suv39h1 peptide.

**Supplementary Table 2. MS analysis of SKP2 deacetylation by SIRT6.** The file includes the following info: (1) Sample peptide MSMS analysis of SKP2 -SIRT6; we detected two acetylated peptides K.S$K_2$acGSD$K_6$acDFVIVR.R on the position range 72-83. (2) Sample peptide MSMS analysis of SKP2 +SIRT6. The two replicas detected the two phosphorilation sites in the same peptide, K.$S_1$phosKG$S_4$phosDKDFVIVR.R serine 72 and serine 75. The peptides were detected by 2 kinds of fragmentation (CID+ETD) but high confidence score.

**Supplementary Table 3. Primers used for cloning**

| | |
|---|---|
| EcoRISirt6U | CCGAATTCATGTCGGTGAATTACGCGGCGGGGCTG |
| SirT6HA-NotI | CCGCGGCCGCGCTGGGGACCGCCTTGGCCT |
| S6H133YL | CCACAAACATGTTCCCGTAGAGCTCTGC |
| S6H133YU | GCAGAGCTCTACGGGAACATGTTTGTGG |
| S6HAG60AU | AGCACTGCCTCTGCCATCCCCGACTTCAGG |
| S6HAG60AL | CCTGAAGTCGGGGATGGCAGAGGCAGTGCT |
| SirT1BamHIU | CCGGATCCATGGCGGACGAGGCGGCCCT |
| SirT1NotIL | CCGCGGCCGCTGATTTGTTTGATGGATAGT |
| mycG9aEcoRIU | AAGAATTCGCAAGCGGCGATGGCGGCGG |
| mycG9aXhoIL | GGCTCGAGCCTGTGTTGACAGGGGGCAGGGA |
| mycGLPXbaIU | CCTCTAGAATGGCCGCCGCCGATGCCGAGGCA |
| mycGLPXbaIL | GGTCTAGACGTAGGGGGTCGGCGGCAGCCGCGG |
| SuvmycSETNotIU | CGGCGGCCGCATCCGATATGACCTCTGCATCT |
| SuvmycSETXhoIL | GGCTCGAGCTAGAAGAGGTATTTGCGGCAGG |
| BamHIdeltaPRESETU | ATCACCCTCAACCAGGGATCCGTGGCTGTGAGTGCCAG |
| BamHIdeltaPRESETL | CTGGCACTCACAGCCACGGATCCCTGGTTGAGGGTGAT |
| BamHIdeltaPRESETfin U | GTAGTCCAGAAAGGCATCGGATCCGATCTCTGCATCTTC |
| BamHIdeltaPRESETfin | GAAGATGCAGAGATCGGATCCGATGCCTTTCTGGACTA |

| | |
|---|---|
| L | C |
| deltaCmycSuv39U | TACAACATGCAAGTGGACTGAGTGGACATGGAGAGTAC C |
| deltaCmycSuv39L | GGTACTCTCCATGTCCACTCAGTCCACTTGCATGTTGTA |
| PRESETmutCAU | CAGCCCATCTACGAGGCCAACTCCCGCGCTTGCTGTGG CTATGACGCCCCAAACCGTGTAGTC |
| PRESETmutCAL | GACTACACGGTTTGGGGCGTCATAGCCACAGCAAGCGC GGGAGTTGGCCTCGTAGATGGGCTG |
| PRESETC818386AU | AACCAGGTAGCTGTTGGCGCTGAGGCCCAGGACGCTCT GTTGGCACCCACT |
| PRESETC818386AL | AGTGGGTGCCAACAGAGCGTCCTGGGCCTCAGCGCCAA CAGCTACCTGGTT |
| PRESETC9495AU | GCACCCACTGGAGGCGCTGCCCCTGGAGCATCCCTGCA C |
| PRESETC9495AL | GTGCAGGGATGCTCCAGGGGCAGCGCCTCCAGTGGGT GC |
| PMSCVmycSuv39h1F | CCCTCGAGGACCGGGGAAAGATGGCGGACG |
| PMSCVmycSuv39h1R | CCGAATTCCTAGAAGAGGTATTTTCGGCAAGC |
| SKP2-S7275DF | CGGCTGAAGGACAAAGGGGACGACAAAGACTTTG |
| SKP2-S7275DR | CAAAGTCTTTGTCGTCCCCTTTGTCCTTCAGCCG |
| SKP2-K717377R-F | ACGGCTGAGAAGCAGAGGGAGTGACAGAGACTTTGTG |
| SKP2-K717377R-R | CACAAAGTCTCTGTCACTCCCTCTGCTTCTCAGCCGT |
| CHIPFLAGEcoRIU | CCGAATTCCATGAAGGGCAAGGAGGAGAAGGA |
| CHIPFLAGBamHIL | CCGGATCCGTAGTCCTCCACCCAGCCATTCTC |
| IKbaBamHIU | CCGGATCCATGTTCCAGGCGGCCGAGCGC |
| IKbaNotIL | GCGGCCGCTAACGTCAGACGCTGGCCTC |
| shSirT6KDF | CCGGAAGAATGTGCCAAGTGTAAGACTCGAGTCTTACAC TTGGCACATTCTTTTTTTG |
| shSirT6KDR | AATTCAAAAAAAGAATGTGCCAAGTGTAAGACTCGAGTC TTACACTTGGCACATTCTT |

**Table S4. Primers used in qPCR.**

| | |
|---|---|
| hIkbalphaF | GATCCGCCAGGTGAAGGG |
| hIkbalphaR | GCAATTTCTGGCTGGTTGG |
| hA20-F | GTCCGGAAGCTTGTGGCGCT |
| hA20-R | CCAAGTCTGTGTCCTGAACGCCC |

| | |
|---|---|
| hIL8-F | GGCAGCCTTCCTGATTTCTG |
| hIL8-R | CTTGGCAAAACTGCACCTTCA |
| hGRO-1 F | AGGAAGCTCACTGGTGGCTG |
| hGRO-1 R | TAGGCACAATCCAGGTGGC |
| hIAP2F | ATGCTTTTGCTGTGATGGTG |
| hIAP2R | TGAACTTGACGGATGAACTCC |
| h14-3-3sigma-F | GTCTGATCCAGAAGGCCAAG |
| h14-3-3sigma-R | CTCCTCGTTGCTTTTCTGCT |
| SUV39h1-fw | GTCATGGAGTACGTGGGAGAG |
| SUV39h1-rev | CCTGACGGTCGTAGATCTGG |
| RT.hHPRT1-F | TGACACTGGCAAAACAATGCA |
| RT.hHPRT1-R | GGTCCTTTTCACCAGCAAGCT |
| RT.hNCL-F | CTGCCTCAGAGGATGAG |
| RT.hNCL-R | TCTGTTTGGCCATTTCCTTC |
| hEEF2 F | TGGAGATCTGCCTGAAGGAC |
| hEEF2 R | GACTTGGAGAGGCAGAGAGCAC |
| MajorsatU | GACGACTTGAAAAATGACGAAATC |
| MajorsatL | CATATTCCAGGTCCTTCAGTGTGC |
| MinorsatU | CATGGAAAATGATAAAAACC |
| MinorsatL | TCTAATATGTTCTACAGTG |
| L1_ORF1 - F | CACTCCCACCCCACCTAGT |
| L1_ORF1 - R | TAACTCTTTAGCAGTGCTCTCCTGT |
| RT.mHPRT1-1F | TCAGTCAACGGGGGACATAAA |
| RT.mHPRT1-1R | GGGGCTGTACTGCTTAACCAG |
| mEEF2 F | TGTCAGTCATCGCCCATGTG |
| mEEF2 R | CATCCTTGCGAGTGTCAGTGA |
| Mrpl38 f | AGGATGCCAAGTCTGTCAAGA |
| Mrpl38 r | TCCTTGTCTGTGATAACCAGG |
| hNFKBIApromoterF | GACGACCCCAATTCAAATCG |
| hNFKBIApromoterR | TCAGGCTCGGGGAATTTCC |

## SUPPLEMENTAL REFERENCES


1. Vaquero, A. et al. Human SirT1 interacts with histone H1 and promotes formation of facultative heterochromatin. *Mol Cell* **16**, 93-105 (2004).

2. Melcher, M. et al. Structure-function analysis of SUV39H1 reveals a dominant role in heterochromatin organization, chromosome segregation, and mitotic progression. *Mol Cell Biol* **20**, 3728-41 (2000).
3. Inuzuka, H. et al. Acetylation-dependent regulation of Skp2 function. *Cell* **150**, 179-93 (2012).
4. Ohta, T., Michel, J.J., Schottelius, A.J. & Xiong, Y. ROC1, a homolog of APC11, represents a family of cullin partners with an associated ubiquitin ligase activity. *Mol Cell* **3**, 535-41 (1999).
5. Cordero-Espinoza, L. & Hagen, T. Regulation of Cullin-RING ubiquitin ligase 1 by Spliceosome-associated protein 130 (SAP130). *Biol Open* **2**, 838-44 (2013).
6. Teixeira, F.R. et al. The F-box protein FBXO25 promotes the proteasome-dependent degradation of ELK-1 protein. *J Biol Chem* **288**, 28152-62 (2013).
7. Ronnebaum, S.M., Wu, Y., McDonough, H. & Patterson, C. The ubiquitin ligase CHIP prevents SirT6 degradation through noncanonical ubiquitination. *Mol Cell Biol* **33**, 4461-72 (2013).
8. Mulero, M.C. et al. Chromatin-bound IkappaBalpha regulates a subset of polycomb target genes in differentiation and cancer. *Cancer Cell* **24**, 151-66 (2013).
9. Boehm, J.S. et al. Integrative genomic approaches identify IKBKE as a breast cancer oncogene. *Cell* **129**, 1065-79 (2007).
10. Di Micco, R. et al. Interplay between oncogene-induced DNA damage response and heterochromatin in senescence and cancer. *Nat Cell Biol* **13**, 292-302 (2011).
11. Sarbassov, D.D., Guertin, D.A., Ali, S.M. & Sabatini, D.M. Phosphorylation and regulation of Akt/PKB by the rictor-mTOR complex. *Science* **307**, 1098-101 (2005).
12. Lu, W. et al. SKP2 inactivation suppresses prostate tumorigenesis by mediating JARID1B ubiquitination. *Oncotarget* **6**, 771-88 (2015).
13. Shevchenko, A., Tomas, H., Havlis, J., Olsen, J.V. & Mann, M. In-gel digestion for mass spectrometric characterization of proteins and proteomes. *Nat Protoc* **1**, 2856-60 (2006).
14. Nielsen, M.L. et al. Iodoacetamide-induced artifact mimics ubiquitination in mass spectrometry. *Nat Methods* **5**, 459-60 (2008).
