# Supplementary material for: SIRT6-dependent cysteine monoubiquitination in the PRE-SET domain of Suv39h1 regulates the NF-κB pathway
Source: Nat Commun. 2018 Jan 9;9:101. doi: 10.1038/s41467-017-02586-x (PMC5760577; doi:10.1038/s41467-017-02586-x)
Supplement: Supplementary file 2 — Descriptions of Additional Supplementary Files [file 41467_2017_2586_MOESM2_ESM.pdf]

Descriptions of Additional Files

File Name: Supplementary Dataset 1
Description: MS analysis of Suv39h1mUb promoted by SIRT6 and SKP2. The file includes cysteine gly-gly signature identified upon SIRT6 (1) or SKP2 incubation (2). The peptides included in both tables represent the detected Suv39h1 peptides identified in the Suv39h1 mUb band upon expression of both proteins. In the same experiment, in the absence of SIRT6 or SKP2 we did not detect any Suv39h1 band corresponding to mUb. Accordingly, MS analysis of the area of the gel equivalent to mUb did not render any Suv39h1 peptide.

File Name: Supplementary Dataset 2
Description: MS analysis of SKP2 deacetylation by SIRT6. The file includes the following info: (1) Sample peptide MSMS analysis of SKP2 -SIRT6; we detected two acetylated peptides K.SK2acGSDK6acDFVIVR.R on the position range 72-83. (2) Sample peptide MSMS analysis of SKP2 +SIRT6. The two replicas detected the two phosphorilation sites in the same peptide, K.S1phosKGS4phosDKDFVIVR.R serine 72 and serine 75. The peptides were detected by 2 kinds of fragmentation (CID+ETD) but high confidence score.
